# A Randomized Trial of BCG to Reduce COVID-19 in Healthcare Workers (BRACE)

**DOI:** 10.1056/NEJMoa2212616

**Published:** 2023-04-27

**Authors:** Laure F. Pittet, Nicole L. Messina, Francesca Orsini, Cecilia L. Moore, Veronica Abruzzo, Simone Barry, Rhian Bonnici, Marc Bonten, John Campbell, Julio Croda, Margareth Dalcolmo, Kaya Gardiner, Grace Gell, Susie Germano, Adriano Gomes-Silva, Casey Goodall, Amanda Gwee, Tenaya Jamieson, Bruno Jardim, Tobias R Kollman, Marcus V.G. Lacerda, Katherine J. Lee, Michaela Lucas, David J. Lynn, Laurens Manning, Helen S. Marshall, Ellie McDonald, Craig F. Munns, Suellen Nicholson, Abby O’Connell, Roberto D. de Oliveira, Susan Perlen, Kirsten P. Perrett, Cristina Prat-Aymerich, Peter C. Richmond, Jesus Rodriguez-Baño, Glauce dos Santos, Patricia V. da Silva, Jia Wei Teo, Paola Villanueva, Adilia Warris, Nicholas J. Wood, Andrew Davidson, Nigel Curtis

**Affiliations:** 1. Infectious Diseases Group, Murdoch Children’s Research Institute, Parkville, Victoria, Australia.; 2. Department of Paediatrics, The University of Melbourne, Parkville, Victoria, Australia.; 3. Infectious Diseases, The Royal Children’s Hospital Melbourne, Parkville, Victoria, Australia.; 4. Infectious Diseases Unit, Department of Paediatrics, Gynaecology and Obstetrics, Faculty of Medicine, University of Geneva and University Hospitals of Geneva, Geneva, Switzerland.; 5. Clinical Epidemiology and Biostatistics Unit, Murdoch Children’s Research Institute, Parkville, Victoria, Australia.; 6. Melbourne Children’s Trial Centre, Murdoch Children’s Research Institute, Parkville, Victoria, Australia; 7. Precision Medicine Theme, South Australian Health and Medical Research Institute, Adelaide, South Australia, Australia.; 8. Department of Thoracic Medicine, Royal Adelaide Hospital, Adelaide, South Australia, Australia.; 9. Julius Center for Health Sciences and Primary Care, University Medical Centre Utrecht, Utrecht University, Utrecht, the Netherlands.; 10. University of Exeter Medical School, Exeter, EX1 2LU, United Kingdom.; 11. Fiocruz Mato Grosso do Sul, Fundação Oswaldo Cruz, Campo Grande, MS, Brazil.; 12. Universidade Federal de Mato Grosso do Sul - UFMS, Campo Grande, MS, Brazil.; 13 Department of Epidemiology of Microbial Diseases, Yale School of Public Health, New Haven, CT, USA.; 14. Helio Fraga Reference Center, Oswaldo Cruz Foundation Ministry of Health, Estrada de Curicica, 2000, 22.780-192, Curicica, Rio de Janeiro, Brazil; 15. Catholic University, Rio de Janeiro, 22541-041, Brazil.; 16. Research Operations, The Royal Children’s Hospital Melbourne, Parkville, Victoria, Australia.; 17. Laboratório Interdisciplinar de Pesquisas Médicas, Instituto Oswaldo Cruz, Fiocruz, Avenida Brasil 4365, 21040 360, Manguinhos, Rio de Janeiro, Brazil.; 18. Institute of Clinical Research Carlos Borborema, Doctor Heitor Vieira Dourado Tropical Medicine Foundation, Manaus, Brazil.; 19. Wesfarmers Centre for Vaccines and Infectious Diseases, Telethon Kids Institute, Nedlands, Western Australia, Australia.; 20. Instituto Leônidas & Maria Deane, Oswaldo Cruz Foundation Ministry of Health, Manaus, Amazonas, Brazil.; 21. University of Texas Medical Branch (UTMB), Galveston, Texas, 77555, USA.; 22. Department of Immunology, Pathwest, QE2 Medical Centre, Nedlands, Western Australia, Australia.; 23. Department of Immunology, Sir Charles Gairdner Hospital, Nedlands, Western Australia, Australia.; 24. Department of Immunology and General Paediatrics, Perth Children’s Hospital, Nedlands, Western Australia, Australia.; 25. School of Medicine, The University of Western Australia, Perth, Western Australia, Australia.; 26. Flinders Health and Medical Research Institute, Flinders University, Bedford Park, South Australia, Australia.; 27. Department of Infectious Diseases, Fiona Stanley Hospital, Murdoch, Western Australia, Australia.; 28. Vaccinology and Immunology Research Trials Unit, Women’s and Children’s Health Network, North Adelaide, South Australia.; 29. Adelaide Medical School and Robinson Research Institute, The University of Adelaide, Adelaide, South Australia.; 30. Department of Endocrinology & Diabetes, The Children’s Hospital at Westmead, Westmead, New South Wales, Australia.; 31. Faculty of Medicine and Health, The University of Sydney, Sydney, New South Wales, Australia.; 32. Victorian Infectious Diseases Reference Laboratory, The Royal Melbourne Hospital, The Peter Doherty Institute for Infection and Immunity, Parkville, Victoria, Australia.; 33. Exeter Clinical Trials Unit, University of Exeter Medical School, Exeter, EX1 2LU, United Kingdom.; 34. Department of Allergy and Immunology, The Royal Children’s Hospital Melbourne, Parkville, Victoria, Australia.; 35. Institut d’Investigació Germans Trias i Pujol, Departament de Genètica i Microbiologia, Universitat Autònoma de Barcelona, CIBER de enfermedades respiratorias (CIBERES), Instituto de Salud Carlos III, Badalona, Barcelona, Spain.; 36. Infectious Diseases and Microbiology division, Hospital Universitario Virgen Macarena, Seville, Spain.; 37. Department of Medicine, University of Seville, Biomedicines Institute of Seville/CSIC, 41009 Seville, Spain.; 38. CIBERINFEC, Instituto de Salud Carlos III, Madrid, Spain.; 39. Medical Research Council Centre for Medical Mycology, University of Exeter, EX4 4QD, UK.; 40. Sydney Children’s Hospitals Network, Westmead, New South Wales, Australia.; 41. National Centre for Immunisation Research and Surveillance of Vaccine Preventable Disease, Westmead, New South Wales, Australia.

## Abstract

**BACKGROUND:**

The bacille Calmette-Guérin (BCG) vaccine has immunomodulatory ‘off-target’ effects hypothesised to protect against COVID-19.

**METHODS:**

In this international, multicentre, double-blind, placebo-controlled trial, healthcare workers were randomised to BCG-Denmark vaccination or saline placebo and followed for 12 months. The primary outcomes of symptomatic and severe COVID-19 were assessed at 6 months.

**RESULTS:**

A total of 3988 participants were randomised; recruitment ceased prior to reaching the planned sample size due to the availability of COVID-19 vaccines. The estimated risk of symptomatic COVID-19 by 6 months was higher in the BCG group (14.7%) compared with the placebo group (12.3%; risk difference +2.4%; 95% confidence interval [CI] −0.7% to +5.5%; p=0.13). The risk of severe COVID-19 by 6 months (comprising mainly those reporting unable to work for ≥3 days) was also higher in the BCG group (7.6%) compared with the placebo group (6.5%; risk difference +1.1%; 95% CI −1.2% to +3.5%; p=0.34). In supplementary and sensitivity analyses using less conservative censoring rules, the risk differences were similar but the confidence intervals narrower. There were 5 hospitalisations due to COVID-19 in each group (including 1 death in the placebo group). The risk of any COVID-19 episode was greater in the BCG group (hazard ratio 1.23; 95% CI 0.96 to 1.59). No safety concerns were identified.

**CONCLUSION:**

BCG-Denmark vaccination did not reduce the likelihood of COVID-19 in healthcare workers.

In addition to protecting against its target disease, tuberculosis, the bacille Calmette-Guérin (BCG) vaccine has immunomodulatory ‘off-target’ effects that may protect against unrelated infections.^1–3^ BCG has been associated with reduced all-cause mortality in infants^4^ and against respiratory infections in adolescents and adults.^5–7^

Early in the SARS-CoV-2 pandemic, it was proposed that BCG vaccine could be repurposed to protect against COVID-19.^8^ It was hypothesised that the immunomodulatory properties of this vaccine might enhance protection against SARS-CoV-2 thus bridging the gap until pathogen-specific vaccines were available.^8,9^

In the *B**CG vaccination to* *R**educe the imp**A**ct of* *C**OVID-19 in h**E**althcare workers* (BRACE) randomised controlled trial (RCT), we aimed to determine if BCG-Denmark vaccination reduces the incidence and severity of COVID-19 in adult healthcare workers compared with placebo.^10^

## METHODS

### TRIAL DESIGN AND SETTING

This multicentre phase 3 RCT in healthcare workers, was done in two stages. Stage 1 (recruitment March 2020 to May 2020) took place in Australia only. Stage 2 (recruitment May 2020 to April 2021) took place in Australia, the Netherlands, Spain, the United Kingdom and Brazil, and was double-blinded and placebo-controlled. In Stage 2, participants were randomised in a 1:1 ratio to receive intradermal BCG-Denmark vaccination or saline placebo and were followed up for 12 months, with the primary outcome assessed at 6 months.^10^ As pre-specified in our statistical analysis plan,^11^ this report focuses only on Stage 2 of the trial as there was negligible SARS-CoV-2 community transmission during Stage l. The protocol has been published^10^ and is available in the Supplementary Appendix.

ClinicalTrials.gov NCT04327206.

### OVERSIGHT

The trial was approved by the ethics committee at each site and overseen by a steering committee and an independent data safety and monitoring board (DSMB). The investigators designed the study. A subgroup of authors collected and analysed the data. The joint first authors and last authors vouch for the accuracy and completeness of the data and for the fidelity of the trial to the protocol, which is available at NEJM.org.

### PARTICIPANTS AND ELIGIBILITY CRITERIA

Potential participants’ eligibility was ascertained during a baseline visit. Exclusion criteria included: previous positive SARS-CoV-2 test; contraindication to BCG vaccine; vaccination with: BCG within the last year, any other live-attenuated vaccine within the last month or any COVID-specific vaccine; and involvement in another COVID-19 prevention trial. All participants had blood samples at baseline for SARS-CoV-2 serology, and those in Brazil also had a baseline SARS-CoV-2 respiratory swab PCR taken. Ethics committee approval was obtained at each site and all participants provided written informed consent.

### RANDOMISATION

The computer-generated randomisation list was prepared by an independent statistician, and we used web-based randomisation, accessed by study staff following consent and baseline assessment. Randomisation was stratified by study region, age (<40 years; 40 to 59 years; ≥60 years) and presence/absence of medical comorbidity. Participants, investigators, outcome assessors, data managers, trial statisticians and trial staff were blinded to the randomisation group throughout the trial. A photo was taken of the injection site ‘bleb’ to confirm correct administration.

### INTERVENTION

A single dose of 0.1 mL BCG-Denmark (AJ Vaccines, Copenhagen; corresponding to 2–8 ×10^5^ colony forming units of *Mycobacterium bovis*, Danish strain 1331) or saline placebo was given as an intradermal injection in the region of the deltoid muscle.

### OUTCOME MEASURES

The trial had two primary outcomes: incidence of ‘symptomatic COVID-19’ and incidence of ‘severe COVID-19’ during the 6 months after randomisation. Complete definitions of primary and secondary outcomes are available in the Supplementary Appendix. Briefly, symptomatic COVID-19 was defined, in accordance with the case definition used internationally at the start of the trial, as an episode of illness with fever or at least one symptom of respiratory disease (including sore throat, cough, and shortness of breath), and evidence of SARS-CoV-2 infection by PCR, rapid antigen test (RAT) or serology. ‘Severe COVID-19’ was defined as an episode of illness with evidence of SARS-CoV-2 infection (PCR, RAT or serology) plus at least one of the following as a consequence of COVID-19: (i) death; (ii) hospitalisation; or (iii) non-hospitalised severe disease, defined, for the purpose of this trial, as being confined to bed or unable to work for ≥3 days.

Secondary outcomes included: time to COVID-19 onset; number of COVID-19 episodes; number of days with symptoms, absence from work or confined to bed; complications (including pneumonia, need for oxygen, hospitalisation, admission to critical care, mechanical ventilation, death); and asymptomatic infection, all within 6 months of randomisation. Vaccine-related adverse reactions were also monitored.

### DATA AND SAMPLE COLLECTION

The REDCap platform was used for data collection.^12^ Participants were asked weekly if they had been unwell using a custom-built smartphone application (Trial Symptom Tracker, WeGuide) and/or by direct contact (phone call, text message). During each episode of illness, symptoms were recorded daily, and participants were asked to undergo SARS-CoV-2 testing. More detailed questionnaires were completed at baseline and 3-monthly during follow-up. Additional information on hospitalisations was obtained from medical records. Blood was collected at baseline, and 3, 6, 9, and 12 months after randomisation for measurement of anti-SARS-CoV-2 nucleocapsid antibodies (Roche Cobas Elecsys anti-SARS-CoV-2 assay).^13^ A biobank of other samples was also established.

### STATISTICAL ANALYSIS

The statistical analysis plan^14^ was finalised and made publicly available before unblinding: full details, including the sample size calculation (n=7244), are available in the Supplementary Appendix. For the primary outcomes, survival analysis (adjusted for stratification factors) was used to estimate the proportion with a COVID-19 episode by 6 months in each group and the risk difference. Follow-up was censored at 6 months, or at time of first COVID-19-specific vaccine, or when it could not be ascertained whether a COVID-19 episode had occurred (missing data for three consecutive days or more, or illness episode without COVID-19 test result). Most analyses were done using a modified intention-to-treat (mITT) population, restricted to participants with a negative baseline SARS-CoV-2 test result.

Pre-planned supplementary analyses were done to provide additional insights: (i) including follow-up time after receipt of a COVID-19-specific vaccine; (ii) excluding episodes starting ≤14 days from randomisation; (iii) censoring participants at any subsequent vaccination (e.g., influenza vaccine); (iv) using the ITT population. Sensitivity analyses were also done: (i) restricted to episodes occurring after BCG/placebo; (ii) using PCR/RAT results only (without serology) for defining COVID-19 episodes (in the ITT population); (iii) using less conservative censoring rules for missing data. Table S1 details the primary, sensitivity and supplementary estimands.

Pre-planned sub-group analyses were done by: (i) age group (<40 years/40 to 59 years/≥60 years); (ii) presence of comorbidities (yes/no, and by comorbidity); (iii) geographical location (Brazil/Europe); (iv) sex (male/female); (v) history of previous BCG (BCG-naïve/previous BCG) for the primary analysis.

## RESULTS

### TRIAL POPULATION

**Figure 1.**
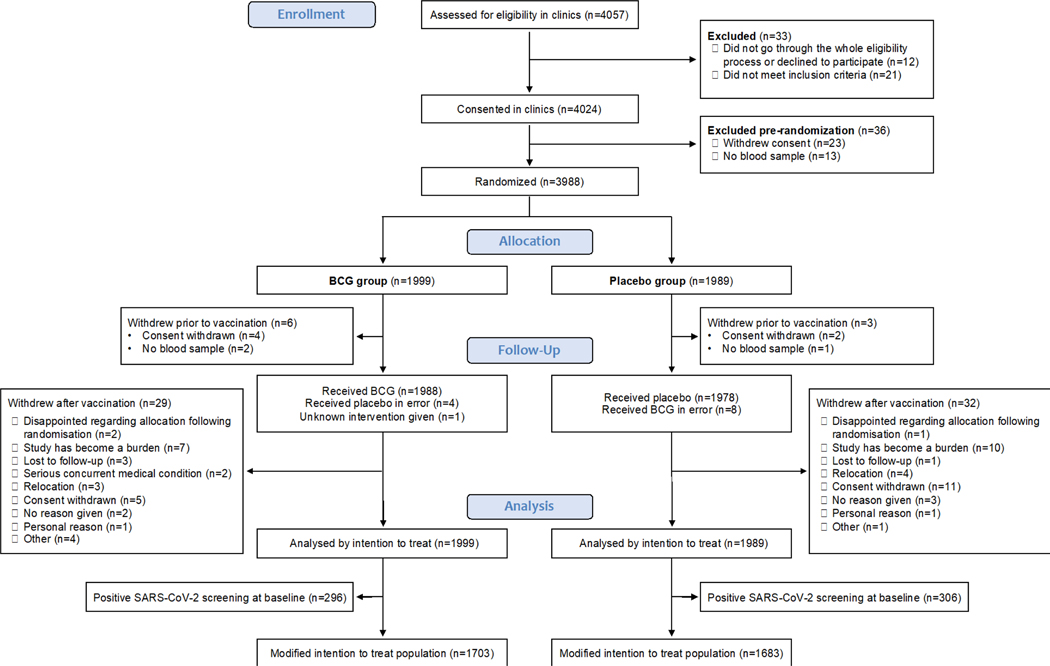
CONSORT diagram. BCG: bacille Calmette-Guérin; SARS-CoV-2: severe acute respiratory syndrome coronavirus 2.

From May 14, 2020 through April 1, 2021, a total of 3988 participants were randomised to BCG (n=1999) or placebo (n=1989) (Figure 1). Recruitment was stopped prematurely prior to reaching the planned sample size due to the global rollout of COVID-19-specific vaccines. The baseline characteristics were similar between the two groups (Tables 1, S2 and S3) apart from a slightly higher proportion of females in the placebo group (75.1% vs 72.3%). Participants were predominately women (73.7%), with a mean age of 42.0 years (standard deviation 12.1 years). A large proportion were enrolled in Brazil (64.4%). Information on the representativeness of the trial participants is provided in Table S20. The baseline SARS-CoV-2 serology was positive in 14.1% of all participants and the baseline SARS-CoV-2 swab was positive in 2.7% of Brazilian participants (and inconclusive or missing in 0.5%). The mITT population consequently included 84.9% of randomised participants (Figure 1): 1703 in the BCG group and 1683 in the placebo group (Tables 1 and S2). Overall, 98% of participants were followed for 6 months or more, with a similar proportion in both groups (Table S5).

### PRIMARY OUTCOMES

**Figure 2.**
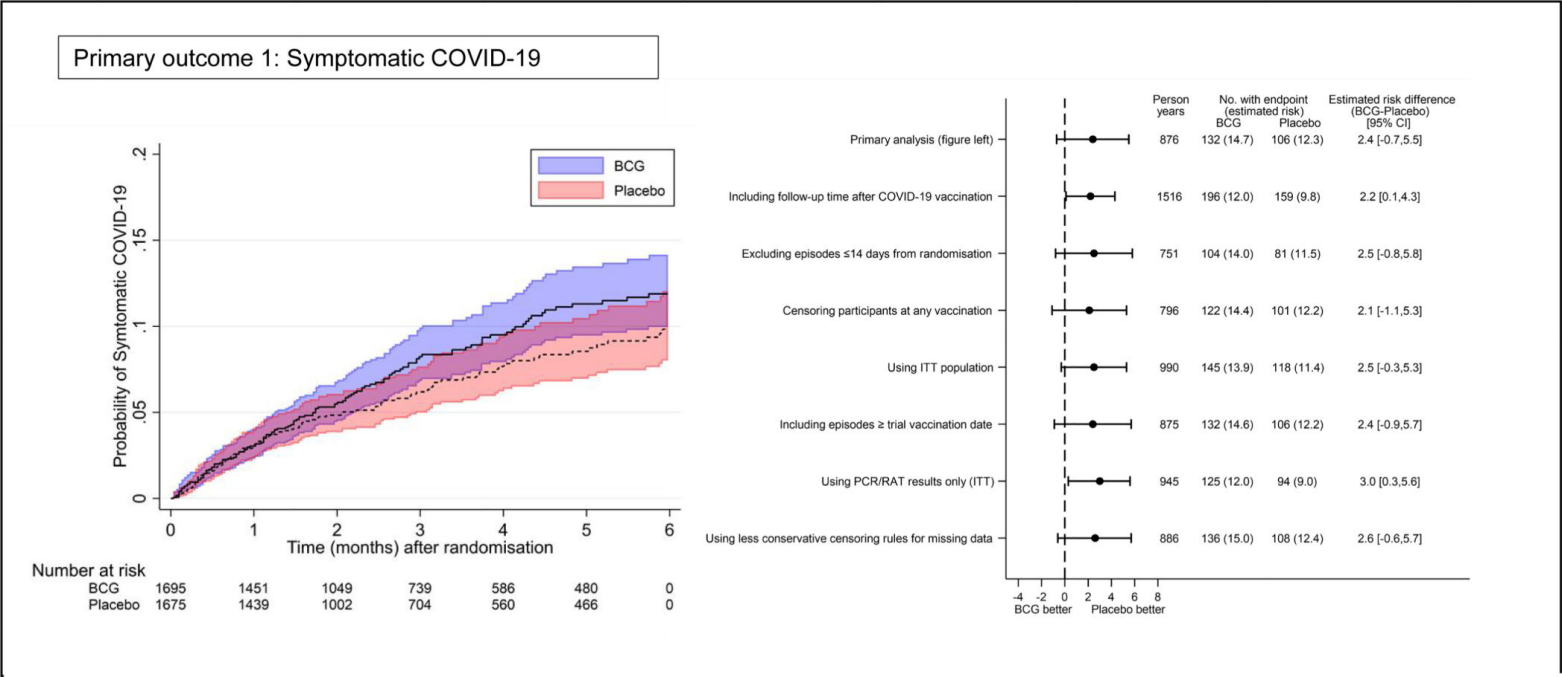

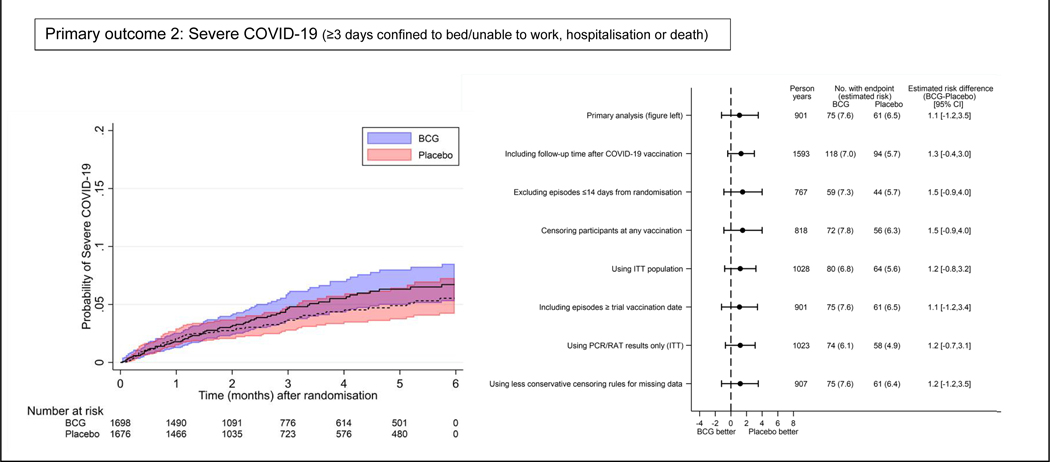
Primary outcomes: primary, sensitivity and supplementary analyses. The primary outcomes of symptomatic (upper panels) and severe (lower panels) COVID-19 are shown using Kaplan-Meier curves with 95% confidence intervals (unadjusted primary analyses; left panels). Forest plots denote difference in percent with symptomatic COVID-19 with 95% confidence intervals (adjusted for stratification factors used in randomisation - primary, sensitivity, and supplementary analyses; right panels). Confidence interval widths have not been adjusted for multiplicity and may not be used in place of hypothesis testing.

In the first 6 months after randomisation, symptomatic COVID-19 occurred in 132 participants in the BCG group (adjusted estimated risk 14.7%) and in 106 participants in the placebo group (12.3%) (difference, +2.4%; 95% confidence interval [CI] −0.7% to +5.5%; p=0.13) and severe COVID-19, as defined in this trial (comprising mainly those reporting unable to work for ≥3 days), occurred in 75 participants in the BCG group (7.6%) and in 61 participants in the placebo group (6.5%) (difference, +1.1%; 95% CI −1.2% to +3.5%; p=0.34) (mITT population; Tables 2, S7 and S8; Figure 2). In supplementary and sensitivity analyses using less conservative censoring rules, the risk differences were similar but the confidence intervals narrower (Figure 2). This included the sensitivity analyses using PCR and RAT only (disregarding serological results), and the analyses ignoring COVID-19-specific vaccination.

### SECONDARY OUTCOMES

The secondary outcomes are presented in Tables 2 and S9 to S17. The probability of any COVID-19 episode within 6 months was greater in the BCG group (adjusted hazard ratio 1.23; 95% CI, 0.96 to 1.59; Table 2 and S9), particularly in the sensitivity analysis relying on PCR and RAT only (adjusted hazard ratio 1.38; 95% CI 1.05 to 1.81), and ignoring COVID-specific vaccination (adjusted hazard ratio 1.24; 95% CI 1.01 to 1.53, Figure S3). There were 5 hospitalisations due to COVID-19 in each group (including 1 death in the placebo group).

When comparing the number of days with symptoms between the BCG and placebo, there was strong evidence of an interaction between treatment arms and two randomisation strata (age group and presence of comorbidities), which rendered an overall comparison between randomisation groups non-interpretable. Post-hoc subgroup analyses revealed that in the ≥60-year age group, the BCG group had fewer days with symptoms compared with the placebo group (32; 95% CI 0.19 to 0.53; Table 2; Figure S4), while no difference between treatment groups was seen in the <40- and 40–59-year age groups. In the subgroup without comorbidities, the BCG group had fewer days with symptoms compared with the placebo group (IRR 0.73; 95% CI 0.58 to 0.91), but the opposite was true in those with comorbidities (IRR 1.49; 95% CI 0.88 to 2.52; Table 2; Figure S4).

### SUBGROUP ANALYSES

**Figure 3.**
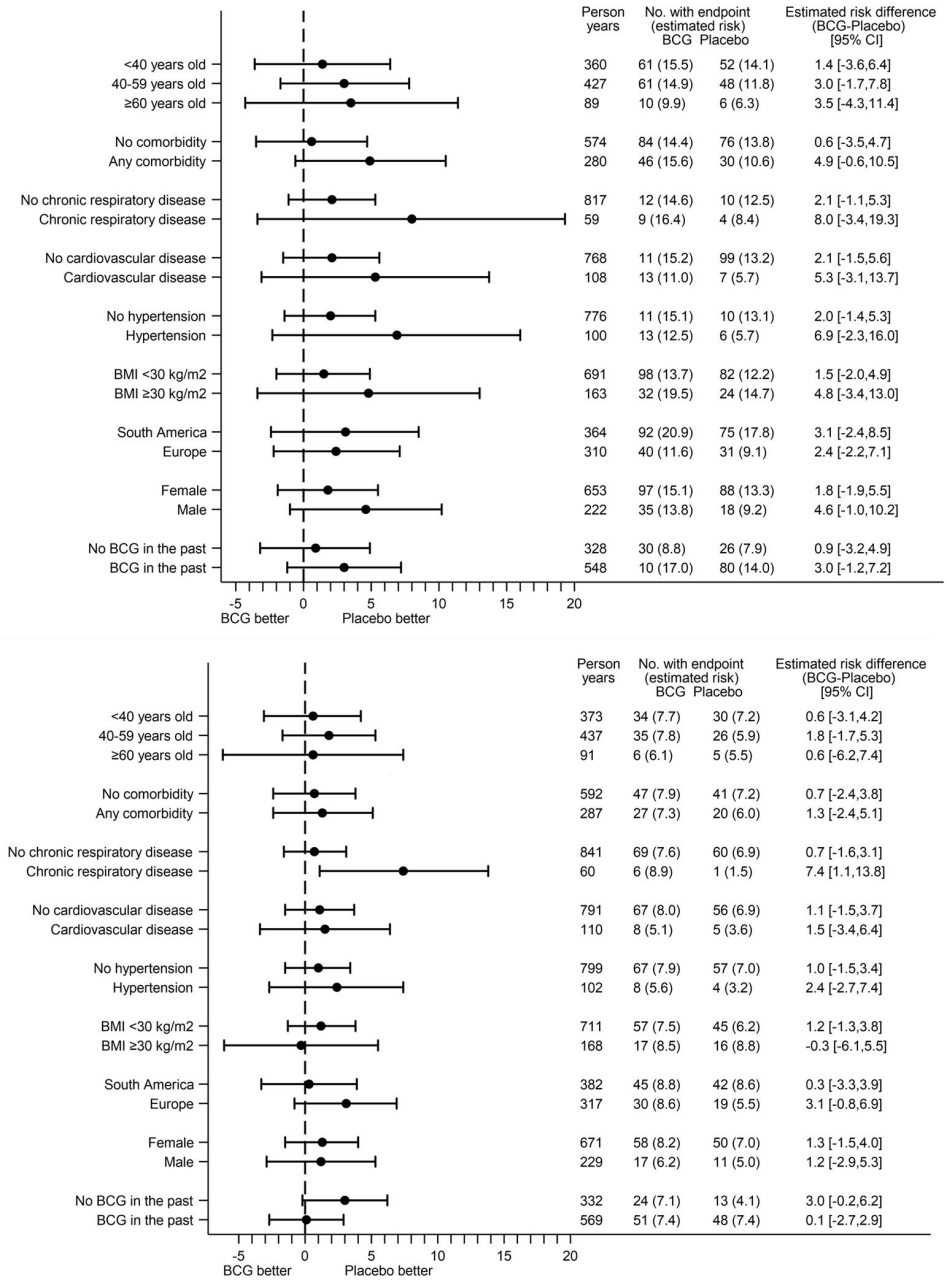
Subgroup analyses. Subgroup analyses of the primary outcomes of symptomatic (upper panel) and severe (lower panel) COVID-19 are shown using Forest plots of the difference in percent and 95% confidence intervals, adjusted for stratification factors used in randomisation. Confidence interval widths have not been adjusted for multiplicity and may not be used in place of hypothesis testing.

In prespecified subgroup analyses, there was little evidence that the treatment effect differed across most of the subgroups (Figure 3). In relation to the influence of previous BCG vaccination, an increase in severe COVID-19 was observed in the BCG group compared with the placebo group in those who were BCG naïve, but not in those who were BCG revaccinated. The probability of symptomatic or severe COVID-19 by 6 months appeared to be slightly higher in the BCG than the placebo group among participants with cardiovascular diseases, and hypertension and chronic respiratory diseases. In the sex-subgroup analysis, although there was minimal evidence for an interaction between sex and the effect of BCG, the disease-free survival curves appeared to separate earlier in the male subgroup than in the female subgroup (Figure S1 and S2).

### SAFETY MONITORING

Details on adverse events are available in the Tables S18 and S19. Briefly, 29 participants reported 30 serious adverse events: 20 in the BCG and 9 in the placebo group. Apart from a painful injection site abscess with lethargy in the BCG group, all were thought unrelated to the intervention by the site investigator.

## DISCUSSION

In this multi-site RCT in healthcare workers in five countries, BCG-Denmark did not reduce the occurrence of COVID-19 within 6 months compared with placebo. It is notable that the risk of an episode of COVID-19 was increased in BCG-vaccinated participants, although the confidence interval around this estimate was wide and crossed zero. In elderly participants and those without a comorbidity, however, episodes were shorter in those in the BCG group.

Previous studies investigating the ability of BCG vaccine to protect against COVID-19 in adults^15–23^ and in animal models^24–27^ have reported conflicting results. Retrospective and ecological studies investigating the association between COVID-19 and BCG vaccination history or national BCG vaccination policy/coverage, are intrinsically limited by many biases, including the long period between BCG vaccination and SARS-CoV-2 exposure.^15^ Trials have also reported conflicting results.^16–23^ In a non-randomised trial of 280 healthcare workers in the United Arab Emirates, of 71 participants who were BCG-Russia revaccinated, none reported COVID-19, compared with 18 of the 209 (8.6%) who declined BCG vaccination.^22^ In contrast, RCTs of BCG vaccination, with the exception of one, have found no protective effect of BCG against COVID-19. In the Greek ACTIVATE-2 RCT in elderly participants (n=301), the cumulative incidence of ‘presumed COVID-19’ was lower following BCG-Moscow compared with placebo, but the primary outcome was defined as possible, probable or definite COVID-19 (without requirement for a positive SARS-CoV-2 test).^16^ When the incidence of PCR-proven COVID-19 cases was assessed, there was no difference between the two groups.^16^ The South-African BCG-CORONA RCT (n=1000) reported a higher risk of severe respiratory tract infections following BCG-Denmark revaccination, compared with placebo, but no impact on COVID-19.^17^ Notably, however, the risk of hospitalisation due to COVID-19 was two-times higher in the BCG group (hazard ratio 2.0; 95% CI, 0.69 to 5.9; p=0.2).^17^ In two Dutch RCTs, BCG-Denmark had no effect on the incidence of COVID-19 episodes (as a secondary outcome) in healthcare workers (n=1511),^18^ or in the elderly (n=2014).^19^ In a Brazilian RCT (n=138), revaccination of healthcare workers with BCG-Moscow did not protect against COVID-19.^20^ Similar findings were reported in a Polish trial (n=354) following revaccination with BCG-Moreau.^21^ Finally, in an ongoing trial investigating the effect of BCG on glycaemic control in type 1 diabetics, over a 15-month period, only 1 of 96 participants who had received 3 doses of BCG-Tokyo (given 2 to 3 years before) had COVID-19 compared with 6 of 48 placebo-vaccinated participants.^23^

The inconsistent results from these and other trials of BCG’s off-target effects are likely explained by a number of factors, including: differing study designs; varying age (infants, adults, elderly), sex distribution and proportion of previously BCG-vaccinated participants; use of different BCG strains (with varying CFUs/dose)^28,29^ and number of doses; and different periods before pathogen exposure, all of which need further investigation. BCG-induced effects might also vary between pathogens. In an animal model, BCG vaccination significantly reduced morbidity and mortality from influenza virus but not from SARS-CoV-2.^25^ Consistent with this, in an RCT of neonatal BCG vaccination, *in vitro* immune responses varied according to pathogen type.^30^ Further, in contrast to previously observed BCG-induced enhancement of *in vitro* cytokine responses to unrelated pathogens,^31,32^ responses to SARS-CoV-2 are decreased by BCG vaccination in adults.^33^ Factors that could have influenced off-target effects of BCG in our trial include the predominance of female participants (in whom off target effects are proposed to differ compared to males) and the underrepresentation of BCG-naïve participants.^34^

Off-target effects of BCG are proposed to, at least in part, be underpinned by epigenetic modifications in immune cells which induce a pro-inflammatory state and stronger cytokine responses to subsequent challenge with unrelated pathogens.^31,32^ Stronger immune responses can be beneficial to clear infections, but might also increase symptoms. Consistent with this, in a human malaria challenge model, BCG-Bulgaria-vaccinated participants had earlier onset and overall more severe clinical symptoms than unvaccinated controls.^35^ The increased risk of symptomatic COVID-19 in the BCG group in our trial might similarly be explained by a BCG-induced more robust immune response. BCG-vaccinated BRACE participants had more activated and effector T cells in response to *in vitro* SARS-CoV-2 stimulation compared with controls.^33^ This might result in more rapid clearance of SARS-CoV-2 leading to a shorter illness. There was some evidence for this in our trial in post-hoc sub-group analyses: the duration of symptoms was lower in the BCG group, though this finding was confined to those over 60-years of age and those without comorbidities.

In our trial, over three-quarters of participants had previously received a BCG vaccine. It has been proposed that the off-target effects of BCG vaccination might be greater in those who have previously received the vaccine compared with those who are BCG-naïve.^36^ However, it is also possible that revaccination does not induce any incremental off-target benefit over that provided by previous BCG vaccination.^37^ It is interesting that in our study, there was weak evidence for an increase in severe COVID-19 in the BCG group, compared with the placebo group, in BCG-naïve but not BCG-revaccinated participants.

Strengths of our trial include its design, larger size, recruitment in 36 sites across 3 continents, blinding of group allocation, stringent COVID-19 case definitions, close active follow-up of participants with daily data collection during illnesses, 3-monthly serology tests, 98% follow-up rate and statistical analysis accounting for COVID-19-specific vaccination.

Main limitations of our trial were the inability to recruit the planned sample size and reduced participant observation time for the primary analysis resulting from the earlier-than-expected availability of COVID-19-specific vaccines. This means the trial was underpowered and susceptible to type II error, and therefore it is possible that BCG increases the risk of COVID-19. Another limitation is that the trial definition of severe COVID-19 differed to that more widely used in COVID-19 studies, which commonly includes only hospitalisations and deaths. Over 90% of participants categorised as severe COVID-19 were captured solely by virtue of being ‘too sick to go to work’ (71%) or ‘unable to get out of bed’ (22%) for ≥3 consecutive days. The effect on severe COVID-19 as more commonly defined by hospitalisation or death could not be meaningfully analysed due to the infrequency of these events. The possibility that BCG induces a more robust immune response that leads to more symptomatic disease, but more rapid clearance of SARS-CoV-2 and consequent reduced hospitalisations and deaths therefore could not be assessed. Another limitation was that our definition of symptomatic COVID-19 was confined to the original case definition which did not include non-febrile episodes without respiratory symptoms. Finally, blinding is a challenge in BCG trials, even with a placebo, due to the injection site reaction that develops in most people. This limitation was mitigated by informing participants that BCG vaccination does not always cause a reaction so that group allocation could not be inferred from the absence of a reaction/scar, using objective primary outcomes and blinding study staff involved with data collection and analysis. However, participant presumption of allocation group might have influenced adherence to COVID-19 control measures, decisions to get influenza or COVID-19-specific vaccines, self-reporting of symptoms or SARS-CoV-2 testing.

In summary, BCG-Denmark vaccination did not reduce, and possibly increased the likelihood of COVID-19 in healthcare workers. Any effect on severe disease as defined by hospitalisation or death could not be assessed. A differential treatment effect of BCG vaccination was found in the duration of COVID-19 episodes in relation to age and presence of comorbidity in post-hoc analyses. It is important that our findings are not extrapolated beyond the effect of BCG-Denmark on COVID-19 in healthcare workers. Several studies report beneficial ‘off-target’ effects of BCG in other situations, particularly infants in high- mortality settings^4^, and ongoing research is attempting to determine potential underlying immunological mechanisms.^38,39^

## Supplementary Material

Supplement

## Figures and Tables

**Table 1.** Participant characteristics.

	ITT		mITT	
		
	BCG	Placebo	BCG	Placebo
	N=1999	N=1989	N=1703	N=1683
Sex, female	1446 (72.3%)	1494 (75.1%)	1245 (73.1%)	1281 (76.1%)
Age, years (mean, SD)	42.0 (12.1)	42.0 (12.1)	42.8 (12.0)	42.8 (12.0)
Any comorbidity	400 (20.0%)	389 (19.6%)	356 (20.9%)	333 (19.8%)
Chronic respiratory disease	126 (6.3%)	108 (5.4%)	111 (6.5%)	92 (5.47%)
Cardiovascular disease / hypertension	261 (13.1%)	250 (12.6%)	233 (13.7%)	214 (12.7%)
Diabetes mellitus	62 (3.1%)	74 (3.7%)	52 (3.1%)	67 (4.0%)
Obesity (BMI ≥30 kg/m^2^)	442 (22.5%) ^a^	417 (21.5%) ^b^	362 (21.7%) ^c^	338 (20.6%) ^d^
Smoker	196 (9.8%)	211 (10.6%)	176 (10.3%)	184 (10.9%)
Previous BCG vaccination	1537 (76.9%)	1521 (76.5%)	1262 (74.1%)	1244 (73.9%)
Positive SARS-CoV-2 serology at baseline	275 (13.8%)	286 (14.4%)	0 (0%)	0 (0%)
Positive SARS-CoV-2 PCR at baseline	34 (2.6%) ^e^	36 (2.8%) ^f^	0 (0%)	0 (0%)
Direct patient contact	1622 (81.1%)	1617 (81.3%)	1363 (80.0%)	1341 (79.7%)
Role				
Nurse / midwife	398 (19.9%)	370 (18.6%)	359 (21.1%)	326 (19.4%)
Medical doctor	208 (10.4%)	197 (9.9%)	197 (11.6%)	187 (11.1%)
Allied Health	384 (19.2%)	392 (19.7%)	329 (19.3%)	339 (20.1%)
Administrative / clerical	307 (15.4%)	303 (15.2%)	257 (15.1%)	252 (15.0%)
Patient service assistant / hospital maintenance	326 (16.3%)	314 (15.8%)	246 (14.4%)	232 (13.8%)
Other	376 (18.8%)	413 (20.8%)	315 (18.5%)	347 (20.6%)
Country / Region				
Australia	216 (10.8%)	206 (10.4%)	214 (12.6%)	206 (12.2%)
Europe / UK	498 (24.9%)	500 (25.1%)	483 (28.4%)	478 (28.4%)
Brazil	1285 (64.3%)	1283 (64.5%)	1006 (59.1%)	999 (59.4%)

Note: Results are number and % with available data unless otherwise stated. Denominators differ for obesity due to missing data

a 1967

b 1941

c 1672

d 1683. Denominators differ as baseline swabs taken in Brazil only

e 1285

f 1283.

**Table 2.** Primary and secondary outcomes.

	BCG	Placebo	Difference	p-value
	N=1703	N=1683	BCG/placebo	
**Primary outcomes**				

**Symptomatic COVID-19 episode by 6 months**	132	106		
Event rate (per 100 person years)	29.4 (24.8; 34.9)	24.4 (20.1; 29.5)		
Unadjusted estimated percent	11.9% (9.9; 13.9)	9.8% (7.9; 11.8)	+2.1% (−0.8; 4.9)	
Adjusted estimated percent ^a^	14.7% (12.0; 17.3)	12.3% (9.7; 14.8)	+2.4% (−0.7; 5.5)	0.13

**Severe COVID-19 episode by 6 months**	75	61		
Death	0	1		
Hospitalised	5	4		
Non-hospitalised severe disease	70	56		
Too sick to get out of bed ^b^	12	18		
Too sick to go to work but not in bed ^b^	58	38		
Event rate (per 100 person years)	16.3 (13.0; 20.5)	13.8 (10.8; 17.8)		
Unadjusted estimated percent	6.7% (5.2; 8.3)	5.5% (4.0; 7.0)	+1.2% (−1.0; 3.4)	
Adjusted estimated percent ^a^	7.6% (5.8; 9.5)	6.5% (4.7; 8.2)	+1.1% (−1.2; 3.5)	0.34

**Secondary outcomes by 6 months**				

Symptomatic and/or severe COVID-19 ^a^	135	107	aHR: 1.23 (0.96; 1.59)	
Pneumonia due to COVID-19 ^a^	7	7	aHR: 0.84 (0.29; 2.41)	
Hospitalisation due to COVID-19 ^a^	5	5	aHR: 0.82 (0.23; 2.87)	
Oxygen therapy due to COVID-19	3 (0.2%)	3 (0.2%)		
Admission to critical care due to COVID-19	2 (0.1%)	2 (0.1%)		
Mechanical ventilation due to COVID-19	2 (0.1%)	1 (0.1%)		
No. of days unable to work due to COVID-19 ^a^	3.0 (0.0; 8.0) ^c^	4.0 (0.0; 11.0) ^c^	aIRR: 0.88 (0.61; 1.26)	
No. of days confined to bed due to COVID-19 ^a^	0.0 (0.0; 1.0) ^c^	0.0 (0.0; 3.0) ^c^	aIRR: 0.76 (0.38; 1.50)	
No. of episodes of COVID-19 due to COVID-19 ^a^	1.0 (1.0; 1.0) ^c^	1.0 (1.0; 1.0) ^c^	aIRR: 0.95 (0.74; 1.22)	
No. of days of unplanned absenteeism ^a^	6.0 (3.0; 11.0) ^d^	6.0 (2.0; 11.0) ^d^	aIRR: 1.12 (0.99; 1.27)	
**Asymptomatic COVID-19**	**12/1071 (1.1%)**	**15/978 (1.5%)**		
Adjusted estimated percent ^a^	1.1% (0.5; 1.8)	1.5% (0.8; 2.3)	−0.4% (−1.4; 0.6)	
**No. of days with symptoms due to COVID-19** ^e^				
*Age group randomisation strata*				
<40 years-old ^f^	15.0 (9.0; 22.0) ^c^	15.0 (11.0; 25.5) ^c^	aIRR: 0.79 (0.61; 1.01)	
40 to 59 years-old ^f^	16.0 (10.0; 23.0) ^c^	14.0 (9.0; 27.0) ^c^	aIRR: 0.92 (0.64; 1.34)	
≥60 years-old ^f^	16.5 (8.0; 24.0) ^c^	38.0 (27.0; 50.0) ^c^	aIRR: 0.32 (0.19; 0.53)	
*Comorbidity randomisation strata*				
Presence of any comorbidity ^f^	19.5 (15.5; 31.5) ^c^	17.0 (12.0; 22.0) ^c^	aIRR: 1.49 (0.88; 2.52)	
Absence of any comorbidity ^f^	13.0 (9.0; 22.0) ^c^	15.5 (11.0; 30.0) ^c^	aIRR: 0.73 (0.58; 0.91)	

Numbers in parentheses represent 95% confidence intervals where not otherwise specified.

Confidence interval widths have not been adjusted for multiplicity and may not be used in place of hypothesis testing.

aHR: adjusted hazard ratio ^a^, aIRR: adjusted incidence rate ratio ^a^.

a Adjusted for stratification factors used at randomisation (age, geographical location, presence of comorbidity).

b For 3 or more consecutive days.

c Within participant with symptomatic and/or severe COVID-19, reported as median (interquartile range).

d Reported as median (interquartile range).

e The number of days with symptoms is presented by age group and presence of comorbidities, due to a significant interaction between treatment arm and age group and presence of comorbidities which rendered the main arm comparison non-interpretable.

f Denominators are: <40 years-old subgroup: 740 BCG, 734 placebo; 40 to 59 years-old subgroup: 811 BCG, 802 placebo; ≥60 years-old subgroup: 152 BCG, 147 placebo; presence of comorbidity subgroup: 356 BCG, 333 placebo; absence of comorbidity subgroup: 1347 BCG, 1350 placebo.

## References

[1] Pollard AJ, Finn A, Curtis N. Non-specific effects of vaccines: plausible and potentially important, but implications uncertain. Arch Dis Child 2017;102(11):1077–1081. DOI: 10.1136/archdischild-2015-310282. 28501809

[2] Fritschi N, Curtis N, Ritz N. Bacille Calmette Guerin (BCG) and new TB vaccines: Specific, cross-mycobacterial and off-target effects. Paediatr Respir Rev 2020;36:57–64. DOI: 10.1016/j.prrv.2020.08.004. 32958428 PMC7439992

[3] Moorlag S, Arts RJW, van Crevel R, Netea MG. Non-specific effects of BCG vaccine on viral infections. Clin Microbiol Infect 2019;25(12):1473–1478. DOI: 10.1016/j.cmi.2019.04.020. 31055165

[4] Higgins JP, Soares-Weiser K, Lopez-Lopez JA, Association of BCG, DTP, and measles containing vaccines with childhood mortality: systematic review. BMJ 2016;355:i5170. DOI: 10.1136/bmj.i5170. 27737834 PMC5063034

[5] Wardhana, Datau EA, Sultana A, Mandang VV, Jim E The efficacy of Bacillus Calmette-Guerin vaccinations for the prevention of acute upper respiratory tract infection in the elderly. Acta Med Indones 2011;43(3):185–90. (https://www.ncbi.nlm.nih.gov/pubmed/21979284). 21979284

[6] Giamarellos-Bourboulis EJ, Tsilika M, Moorlag S, Activate: randomized clinical trial of BCG vaccination against infection in the elderly. Cell 2020;183(2):315–323 e9. (In eng). DOI: 10.1016/j.cell.2020.08.051. 32941801 PMC7462457

[7] Nemes E, Geldenhuys H, Rozot V, Prevention of M. tuberculosis Infection with H4:IC31 vaccine or BCG revaccination. N Engl J Med 2018;379(2):138–149. DOI: 10.1056/NEJMoa1714021. 29996082 PMC5937161

[8] Curtis N, Sparrow A, Ghebreyesus TA, Netea MG. Considering BCG vaccination to reduce the impact of COVID-19. Lancet 2020;395(10236):1545–1546. (In eng). DOI: 10.1016/S0140-6736(20)31025-4. 32359402 PMC7252177

[9] Netea MG, Giamarellos-Bourboulis EJ, Domínguez-Andrés J, Trained immunity: a tool for reducing susceptibility to and the severity of SARS-CoV-2 infection. Cell 2020;181(5):969–977. (In eng). DOI: 10.1016/j.cell.2020.04.042. 32437659 PMC7196902

[10] Pittet LF, Messina NL, Gardiner K, BCG vaccination to reduce the impact of COVID-19 in healthcare workers: Protocol for a randomised controlled trial (BRACE trial). BMJ Open 2021;11(10):e052101. DOI: 10.1136/bmjopen-2021-052101. PMC8557250 34711598

[11] Orsini F BRACE Statistical Analysis Plan for Interim Analysis. Murdoch Childrens Research Institute. 03/06/2021 (https://mcri.figshare.com/articles/online_resource/BRACE_Statistical_Analysis_Plan_for_Interim_Analysis/14721309).

[12] Harris PA, Taylor R, Thielke R, Payne J, Gonzalez N, Conde JG. Research electronic data capture (REDCap) - a metadata-driven methodology and workflow process for providing translational research informatics support. J Biomed Inform 2009;42(2):377–81. DOI: 10.1016/j.jbi.2008.08.010. 18929686 PMC2700030

[13] Bond KA, Williams E, Nicholson S, Longitudinal evaluation of laboratory-based serological assays for SARS-CoV-2 antibody detection. Pathology 2021;53(6):773–779. (In eng). DOI: 10.1016/j.pathol.2021.05.093. 34412859 PMC8289701

[14] Orsini F BRACE Trial SAP. Murdoch Childrens Research Institute. 29/08/2022 (https://mcri.figshare.com/articles/online_resource/BRACE_Trial_SAP/19836157).

[15] Khera D, Chugh A, Khasbage S, Singh S. Does bacille Calmette-Guérin vaccination provides protection against COVID-19: a systematic review and meta-analysis. Indian J Community Med 2021;46(4):592–599. (In eng). DOI: 10.4103/ijcm.IJCM_952_20. 35068716 PMC8729290

[16] Tsilika M, Taks E, Dolianitis K, ACTIVATE-2: A double-blind randomized trial of BCG vaccination against COVID-19 in individuals at risk. Front Immunol 2022;13:873067. (In eng). DOI: 10.3389/fimmu.2022.873067. PMC9294453 35865520

[17] Upton CM, van Wijk RC, Mockeliunas L, Safety and efficacy of BCG revaccination in relation to COVID-19 morbidity in healthcare workers: A double-blind, randomised, controlled, phase 3 trial. EClinicalMedicine 2022;48:101414. (In eng). DOI: 10.1016/j.eclinm.2022.101414. PMC9098089 35582122

[18] Doesschate TT, van der Vaart TW, Debisarun PA, BCG vaccine to reduce healthcare worker absenteeism in COVID-19 pandemic, a randomized controlled trial. Clin Microbiol Infect 2022 (In eng). DOI: 10.1016/j.cmi.2022.04.009. PMC9046133 35489606

[19] Moorlag SJCFM, Taks E, ten Doesschate T, Efficacy of BCG vaccination against respiratory tract infections in older adults during the coronavirus disease 2019 pandemic. Clinical Infectious Diseases 2022. DOI: 10.1093/cid/ciac182. PMC8903481 35247264

[20] Dos Anjos LRB, da Costa AC, Cardoso A, Efficacy and safety of BCG revaccination with M. bovis BCG Moscow to prevent COVID-19 infection in health care workers: a randomized phase II clinical trial. Front Immunol 2022;13:841868. (In eng). DOI: 10.3389/fimmu.2022.841868. PMC8981724 35392074

[21] Czajka H, Zapolnik P, Krzych Ł, A multi-center, randomised, double-blind, placebo-controlled phase III clinical trial evaluating the impact of BCG re-vaccination on the incidence and severity of SARS-CoV-2 infections among symptomatic healthcare professionals during the COVID-19 pandemic in Poland-first results. Vaccines (Basel) 2022;10(2) (In eng). DOI: 10.3390/vaccines10020314. PMC8879775 35214772

[22] Amirlak L, Haddad R, Hardy JD, Khaled NS, Chung MH, Amirlak B. Effectiveness of booster BCG vaccination in preventing COVID-19 infection. Hum Vaccin Immunother 2021;17(11):3913–3915. (In eng). DOI: 10.1080/21645515.2021.1956228. 34403297 PMC8425429

[23] Faustman DL, Lee A, Hostetter ER, Multiple BCG vaccinations for prevention of COVID-19 and other infectious diseases in Type 1 diabetes. Cell Rep Med. DOI: 10.1016/j.xcrm.2022.100728. PMC9376308 36027906

[24] Hilligan KL, Namasivayam S, Clancy CS, Intravenous administration of BCG protects mice against lethal SARS-CoV-2 challenge. J Exp Med 2022;219(2) (In eng). DOI: 10.1084/jem.20211862. PMC8669500 34889942

[25] Kaufmann E, Khan N, Tran KA, BCG vaccination provides protection against IAV but not SARS-CoV-2. Cell Rep 2022;38(10):110502. (In eng). DOI: 10.1016/j.celrep.2022.110502. PMC8858710 35235831

[26] White AD, Sibley L, Sarfas C, Influence of aerosol delivered BCG vaccination on immunological and disease parameters following SARS-CoV-2 challenge in rhesus macaques. Front Immunol 2021;12:801799. (In eng). DOI: 10.3389/fimmu.2021.801799. PMC8863871 35222355

[27] Counoupas C, Johansen MD, Stella AO, A single dose, BCG-adjuvanted COVID-19 vaccine provides sterilising immunity against SARS-CoV-2 infection. NPJ Vaccines 2021;6(1):143. (In eng). DOI: 10.1038/s41541-021-00406-4. 34848711 PMC8633321

[28] Ritz N, Hanekom WA, Robins-Browne R, Britton WJ, Curtis N. Influence of BCG vaccine strain on the immune response and protection against tuberculosis. FEMS Microbiol Rev 2008;32(5):821–41. DOI: 10.1111/j.1574-6976.2008.00118.x. 18616602

[29] Ritz N, Dutta B, Donath S, The influence of bacille Calmette-Guerin vaccine strain on the immune response against tuberculosis: a randomized trial. Am J Respir Crit Care Med 2012;185(2):213–22. DOI: 10.1164/rccm.201104-0714OC. 22071384

[30] Freyne B, Messina NL, Donath S, Neonatal BCG Vaccination Reduces Interferon-gamma Responsiveness to Heterologous Pathogens in Infants From a Randomized Controlled Trial. J Infect Dis 2020;221(12):1999–2009. DOI: 10.1093/infdis/jiaa030. 31990350 PMC7289544

[31] Netea MG, Joosten LA, Latz E, Trained immunity: A program of innate immune memory in health and disease. Science 2016;352(6284):aaf1098. DOI: 10.1126/science.aaf1098. PMC5087274 27102489

[32] Netea MG, Quintin J, van der Meer JW. Trained immunity: a memory for innate host defense. Cell Host Microbe 2011;9(5):355–61. DOI: 10.1016/j.chom.2011.04.006. 21575907

[33] Messina NL, Germano S, McElroy R, Off-target effects of bacillus Calmette-Guérin vaccination on immune responses to SARS-CoV-2: implications for protection against severe COVID-19. Clin Transl Immunology 2022;11(4):e1387. (In eng). DOI: 10.1002/cti2.1387. PMC9028103 35573165

[34] Flanagan KL, Plebanski M. Sex-differential heterologous (non-specific) effects of vaccines: an emerging public health issue that needs to be understood and exploited. Expert Rev Vaccines 2017;16(1):5–13. DOI: 10.1080/14760584.2016.1203260. 27362915

[35] Walk J, de Bree LCJ, Graumans W, Outcomes of controlled human malaria infection after BCG vaccination. Nat Commun 2019;10(1):874. (In eng). DOI: 10.1038/s41467-019-08659-3. 30787276 PMC6382772

[36] Aaby P, Netea MG, Benn CS. Beneficial non-specific effects of live vaccines against COVID-19 and other unrelated infections. Lancet Infect Dis 2023;23(1):e34–e42. DOI: 10.1016/S1473-3099(22)00498-4. 36037824 PMC9417283

[37] Debisarun PA, Kilic G, de Bree LCJ, The impact of BCG dose and revaccination on trained immunity. Clin Immunol 2022;246:109208. DOI: 10.1016/j.clim.2022.109208. 36565972

[38] Prentice S, Nassanga B, Webb EL, BCG-induced non-specific effects on heterologous infectious disease in Ugandan neonates: an investigator-blind randomised controlled trial. Lancet Infect Dis 2021;21(7):993–1003. DOI: 10.1016/S1473-3099(20)30653-8. 33609457 PMC8222005

[39] Bannister S, Kim B, Dominguez-Andres J, Neonatal BCG vaccination is associated with a long-term DNA methylation signature in circulating monocytes. Sci Adv 2022;8(31):eabn4002. DOI: 10.1126/sciadv.abn4002. PMC9355358 35930640

