# Supplementary material for: A Randomized Trial of BCG to Reduce COVID-19 in Healthcare Workers (BRACE)
Source: N Engl J Med. Author manuscript; Available in PMC 2023 Oct 6. (PMC10497190; doi:10.1056/NEJMoa2212616)
Supplement: Supplement [file NIHMS1874591-supplement-Supplement.pdf]

Supplementary Appendix

This appendix has been provided by the authors to give readers additional information about their work.

Contents

BRACE TRIAL CONSORTIUM GROUP 3
RECRUITING CENTRES (SITE LEADS) 7
STATISTICAL ANALYSIS 8
FIGURES AND TABLES 10
SAFETY REPORT 41
REPRESENTATIVENESS OF STUDY PARTICIPANTS 45

Australia (Victoria)

MCRI Central Team: **Prof Nigel Curtis**, Prof Andrew Davidson, Kaya Gardiner, A/Prof Amanda Gwee, Tenaya Jamieson, Dr Nicole Messina, Thilanka Morawakage, Dr Susan Perlen, A/Prof Kirsten Perrett, Dr Laure Pittet, Amber Sastry, Jia Wei Teo;

Biostatisticians: **Francesca Orsini**, Prof Katherine Lee, Dr Cecilia Moore, Suzanna Vidmar;

Data Team: **Dr Laure Pittet**, Rashida Ali, Ross Dunn, Peta Edler, Grace Gell, Casey Goodall, Richard Hall, Ann Krastev, Dr Nathan La, Dr Ellie McDonald, Nick McPhate, Thao Nguyen, Jack Ren, Luke Stevens;

Laboratory Core Team: **Dr Nicole Messina**, Ahmed Alamrousi, Rhian Bonnici, Dr Thanh Dang, Susie Germano, Jenny Hua, Rebecca McElroy, Monica Razmovska, Scott Reddiex, Xiaofang Wang; Laboratory Scientists: Jeremy Anderson, Kristy Azzopardi, Vicki Bennett- Wood, Anna Czajko, Nadia Mazarakis, Conor McCafferty, Frances Oppedisano, Belinda Ortika, Casey Pell, Leena Spry, Ryan Toh, Sunitha Velagapudi, Amanda Vlahos, Ashleigh Wee-Hee; Biobanking: **Pedro Ramos**, Karina De La Cruz, Dinusha Gamage, Anushka Karunanayake, Isabella Mezzetti, Dr Benjamin Ong, Ronita Singh, Enoshini Sooriyarachchi;

Serology testing (VIDRL): **Dr Suellen Nicholson**, Natalie Cain, Rianne Brizuela, Han Huang;

Study Visit and Phone Call Team: **Veronica Abruzzo**, Morgan Bealing, Patricia Bimboese, Kirsty Bowes, Emma Burrell, Dr Joyce Chan, Jac Cushnahan, Hannah Elborough, Olivia Elkington, Kieran Fahey, Monique Fernandez, Catherine Flynn, Sarah Fowler, Marie Gentile Andrit, Bojana Gladanac, Catherine Hammond, Norine Ma, Sam Macalister, Emmah Milojevic, Jesutofunmi Mojeed, Jill Nguyen, Liz O'Donnell, Nadia Olivier, Isabelle Ooi, Stephanie Reynolds, Lisa Shen, Barb Sherry, Judith Spotswood, Jamie Wedderburn, Angela Younes;

Pharmacy Team: **Donna Legge,** Jason Bell, Jo Cheah, Annie Cobbledick, Kee Lim;

Immunisation Team: **Sonja Elia**, Lynne Addlem, Anna Bourke, Clare Brophy, Nadine Henare, Narelle Jenkins, Francesca Machingaifa, Skye Miller, Kirsten Mitchell, Sigrid Pitkin, Kate Wall;

Safety and Quality Monitoring Team: **Dr Paola Villanueva**, A/Prof Nigel Crawford, Dr Laure Pittet, Dr Wendy Norton;

Epworth Healthcare: **Dr Niki Tan**, Thilakavathi Chengodu, Diane Dawson, Victoria Gordon;

Monash Health: **Tony Korman**, Jess O'Bryan, Veronica Abruzzo;

MCRI Start-up Support Team: Sophie Agius, Dr Samantha Bannister, Jess Bucholc, Alison Burns, Beatriz Camesella, Prof John Carlin, Marianna Ciaverella, Maxwell Curtis, Stephanie Firth, Dr Christina Guo, Matthew Hannan, Erin Hill, Sri Joshi, Katherine Lieschke, Megan Mathers, Sasha Odoi, Ashleigh Rak, Dr Chris Richards, Leah Steve, Carolyn Stewart, Dr Eva Sudbury, Helen Thomson, Emma Watts, Fiona Williams, Angela Young;

Legal: **Penny Glenn**, Andrew Kaynes, Amandine Philippart De Floy App Development: Sandy Buchanan, Thijs Sondag, Ivy Xie;

Media and Communications: **Harriet Edmund**, Bridie Byrne, Tom Keeble, Belle Ngien, Fran Noonan, Michelle Wearing-Smith;

Orygen Volunteers: Alison Clarke, Pemma Davies, Oliver Eastwood, Alric Ellinghaus, Rachid Ghieh, Zahra Hilton, Emma Jennings, Athina Kakkos, Iris Liang, Katie Nicol, Sally O'Callaghan, Helen Osman, Gowri Rajaram, Sophia Ratcliffe, Victoria Rayner, Ashleigh Salmon, Angela Scheppokat, Aimee Stevens, Rebekah Street, Nicholas Toogood.

Australia (New South Wales)

Westmead Children's Hospital: **A/Prof Nicholas Wood**, Twinkle Bahaduri, Therese Baulman, Jennifer Byrne, Candace Carter, Mary Corbett, Aiken Dao, Maria Desylva, Dr Andrew Dunn, Evangeline Gardiner, Rosemary Joyce, Dr Rama Kandasamy, Prof Craig Munns, Lisa Pelayo, Dr Ketaki Sharma, Katrina Sterling, Caitlin Uren; Westmead Hospital: Clinton Colaco, A/Prof Mark Douglas, Kate Hamilton; Sydney Children's Hospital: Dr Adam Bartlett, Dr Brendan McMullan, Dr Pamela Palasanthiran, Dr Phoebe Williams; Prince of Wales Hospital: Dr Justin Beardsley, Nikki Bergant, Renier Lagunday, Dr Kristen Overton, Prof Jeffrey Post; St Vincent's Hospital Sydney: Dr Yasmeen Al-Hindawi, Sarah Barney, A/Prof Anthony Byrne, Lee Mead, Marshall Plit.

Australia (South Australia)

SAHMRI: **Prof. David Lynn**, Saoirse Benson, Dr Stephen Blake, Rochelle Botten, Tee Yee Chern, Georgina Eden, Liddy Griffith, Jane James, Dr Miriam Lynn, Angela Markow, Domenic Sacca, Dr Natalie Stevens, Prof. Steve Wesselingh; Royal Adelaide Hospital: Catriona Doran, Dr Simone Barry, Dr Alice Sawka; Women's and Children's Hospital: Dr Sue Evans, Louise Goodchild, Christine Heath, Meredith Krieg, Prof. Helen Marshall, Mark McMillan, Mary Walker.

Australia (Western Australia)

Perth Children's Hospital/Telethon Kids Institute: **Prof Peter Richmond**, Nelly Amenyogbe, Christina Anthony, Annabelle Arnold, Beth Arrowsmith, Rym Ben-Othman, Sharon Clark, Jemma Dunnill, Nat Eiffler, Krist Ewe, Carolyn Finucane, Lorraine Flynn, Camille Gibson, Lucy Hartnell, Elysia Hollams, Heidi Hutton, Lance Jarvis, Jane Jones, Jan Jones, Karen Jones, Jennifer Kent, Prof Tobias Kollmann, Debbie Lalich, Wenna Lee, Rachel Lim, Sonia McAlister, Fiona McDonald, Andrea Meehan, Asma Minhaj, Lisa Montgomery, Melissa O'Donnell, Jaslyn Ong, Joanne Ong, Kimberley Parkin, Glady Perez, Catherine Power, Shadie Rezazadeh, Holly Richmond, Sally Rogers, Nikki Schultz, Margaret Shave, Patrycja Skut, Lisa Stiglmayer, Alexandra Truelove, Dr Ushma Wadia, Rachael Wallace, Justin Waring; Fiona Stanley Hospital: Michelle England, Erin Latkovic, A/Prof Laurens Manning; Sir Charles Gardiner: Dr Susan Herrmann, Prof Michaela Lucas.

Brazil (Manaus)

Manaus: **Dr Marcus Lacerda**, Paulo Henrique Andrade, Fabiane Bianca Barbosa, Dayanne Barros, Larissa Brasil, Ana Greyce Capella, Ramon Castro, Erlane Costa, Dilcimar de Souza, Maianne Dias, José Dias, Klenilson Ferreira, Paula Figueiredo, Thamires Freitas, Ana Carolina Furtado, Larissa Gama, Vanessa Godinho, Cintia Gouy, Daniele Hinojosa, Dr Bruno Jardim, Dr Tyane Jardim, Joel Junior, Augustto Lima, Bernardo Maia, Adriana Marins, Kelry Mazurega, Tercilene Medeiros, Rosangela Melo, Marinete Moraes, Elizandra Nascimento, Juliana Neves, Maria Gabriela Oliveira, Thais Oliveira, Ingrid Oliveira, Arthur Otsuka, Rayssa Paes, Handerson Pereira, Gabrielle Pereira, Christiane Prado, Evelyn Queiroz, Laleyska Rodrigues, Bebeto Rodrigues, Dr Vanderson Sampaio, Anna Gabriela Santos, Daniel Santos, Tilza Santos, Evelyn Santos, Ariandra Sartim, Ana Beatriz Silva, Juliana Silva, Emanuelle Silva, Mariana Simão, Caroline Soares, Antonny Sousa, Alexandre Trindade, Dr Fernando Val, Adria Vasconcelos, Heline Vasconcelos.

Brazil (Mato Grosso do Sul)

Mato Grosso do Sul: **Prof Julio Croda**, Carolinne Abreu, Katya Martinez Almeida, Camila Bitencourt de Andrade, Jhenyfer Thalyta Campos Angelo, Ghislaine Gonçalvez de Araújo Arcanjo, Bianca Maria Silva Menezes Arruda, Wellyngthon Espindola Ayala, Adelita Agripina Refosco Barbosa, Felipe Zampieri Vieira Batista, Fabiani de Morais Batista, Miriam de Jesus Costa, Dr Mariana Garcia Croda, Lais Alves da Cruz, Roberta Carolina Pereira Diogo, Rodrigo Cezar Dutra Escobar, Iara Rodrigues Fernandes, Leticia Ramires Figueiredo, Leandro Galdino Cavalcanti Gonçalves, Sarita Lahdo, Joyce dos Santos Lencina, Guilherme Teodoro de Lima, Larissa Santos Matos, Bruna Tayara Leopoldina Meireles, Debora Quadros Moreira, Lilian Batista Silva Muranaka, Adriely de Oliveira, Karla Regina Warszawski de Oliveira, Matheus Vieira de Oliveira, Prof Roberto Dias de Oliveira, Andrea Antonia Souza de Almeida dos Reis Pereira, Marco Puga, Caroliny Veron Ramos, Thaynara Haynara Souza da Rosa, Karla Lopes dos Santos, Claudinalva Ribeiro dos Santos, Dyenyffer Stéffany Leopoldina dos Santos, Karina Marques Santos, Paulo César Pereira da Silva, Paulo Victor Rocha da Silva, Débora dos Santos Silva, Patricia Vieira da Silva, Bruno Freitas da Rosa Soares, Mariana Gazzoni Sperotto, Mariana Mayumi Tadokoro, Daniel Tsuha, Hugo Miguel Ramos Vieira.

Brazil (Rio de Janeiro)

Rio de Janeiro: **Prof Margareth Maria Pretti Dalcolmo**, Cíntia Maria Lopes Alves da Paixão, Gabriela Corrêa E Castro, Simone Silva Collopy, Renato da Costa Silva, Samyra Almeida da Silveira, Alda Maria Da-Cruz, Alessandra Maria da Silva Passos de Carvalho, Rita de Cássia Batista, Maria Luciana Silva De Freitas, Aline Gerhardt de Oliveira Ferreira, Ana Paula Conceição de Souza, Paola Cerbino Doblas, Ayla Alcoforado da Silva dos Santos, Vanessa Cristine de Moraes dos Santos, Glauce Dos Santos, Dayane Alves dos Santos Gomes, Anderson Lage Fortunato, Adriano Gomes-Silva, Monique Pinto Gonçalves, Paulo Leandro Garcia Meireles Junior, Estela Martins da Costa Carvalho, Fernando do Couto Motta, Ligia Maria Olivo de Mendonça, Girlene dos Santos Pandine, Rosa Maria Plácido Pereira, Ivan Ramos Maia, Jorge Luiz da Rocha, João Victor Paiva Romano, Erica Fernandes da Silva, Marilda Agudo Mendonça Teixeira de Siqueira, Ágatha Cristinne Prudêncio Soares.

The Netherlands

UMC Utrecht: **Prof Marc Bonten,** Sandra Franch Arroyo, A/Prof Cristina Prat Aymerich, Henny Ophorst-den Besten, Anna Boon, Karin M Brakke, Axel Janssen, Marijke A.H. Koopmans, Toos Lemmens, Titia Leurink, Engelien Septer-Bijleveld, Kimberly Stadhouders, Dr Darren Troeman, Marije van der Waal, Marjoleine van Opdorp, Nicolette van Sluis, Beatrijs Wolters; Amphia Hospital: Prof Jan Kluytmans, Jannie Romme, Dr Wouter van den Bijllaardt, Linda van Mook, Dr M.M.L (Miranda) van Rijen; Rijnstate Hospital: P. M. G. Filius, Jet Gisolf, Frances Greven, Danique Huijbens, Dr Robert Jan Hassing, R. C. Pon, Lieke Preijers, J. H. van Leusen, Harald Verheij; Noord West Ziekenhuis: Dr Wim Boersma, Evelien Brans, Paul Kloeg, Kitty Molenaar-Groot, Nhat Khanh Nguyen, Dr Nienke Paternotte, Anke Rol, Lida Stooper; Radboud UMC: Helga Dijkstra, Esther Eggenhuizen, Lucas Huijs, Dr Simone Moorlag, Prof Mihai Netea, Eva Pranger, Dr Esther Taks, Dr Jaap ten Oever, Rob ter Heine; St Antonius Hospital: Kitty Blauwendraat, Dr Bob Meek, Isil Erkaya, Houda Harbech, Dr Nienke Roescher, Rifka Peeters, Menno te Riele, Carmen Zhou.

Spain

Mutua Terrassa University Hospital: Dr Esther Calbo, Cristina Badia Marti, Emma Triviño Palomares, Tomás Perez Porcuna; University Hospital Germans Trias I Pujol: Anabel Barriocanal, Ana Maria Barriocanal, Irma Casas, Jose Dominguez, Maria Esteve, Alicia Lacoma, Irene Latorre, Gemma Molina, Barbara Molina, Dr Antoni Rosell, Sandra Vidal; Hospital Virgen Macarena: Lydia Barrera, Natalia Bustos, Ines Portillo Calderón, David Gutierrez Campos, Jose Manuel Carretero, Angel Dominguez Castellano, Renato Compagnone, Encarnacion Ramirez de Arellano, Almudena de la Serna, Maria Dolores del Toro Lopez, Marie-Alix Clement Espindola, Ana Belen Martin Gutierrez, Alvaro Pascual Hernandez, Virginia Palomo Jiménez, Elisa Moreno, Nicolas Navarrete, Teresa Rodriguez Paño, Prof Jesús Rodríguez-Baño, Enriqueta Tristán, Maria Jose Rios Villegas; University Hospital Cruces: Atsegiñe Canga Garces, Erika Castro Amo, Raquel Coya Guerrero, Dr.Josune Goikoetxea, Leticia Jorge, Cristina Perez; Marqués de Valdecilla University Hospital: Dr María Carmen Fariñas Álvarez, Manuel Gutierrez Cuadra, Dr Francisco Arnaiz de las Revillas Almajano, Pilar Bohedo Garcia, Dr Teresa Giménez Poderos, Claudia González Rico, Blanca Sanchez, Olga Valero, Noelia Vega.

United Kingdom

University of Exeter/Exeter Clinical Trials Unit: **Prof John Campbell,** Anna Barnes, Dr Helen Catterick, Tim Cranston, Phoebe Dawe, Emily Fletcher, Liam Fouracre, Dr Alison Gifford, John Kirkwood, Dr Christopher Martin, Dr Amy McAndrew, Marcus Mitchell, Georgina Newman, Dr Abby O'Connell, Jakob Onysk, Lynne Quinn, Dr Shelley Rhodes, Samuel Stone, Dr Lorrie Symons, Harry Tripp, Prof Adilia Warris, Darcy Watkins, Bethany Whale; St Leonard's Practice: Dr Alex Harding, Gemma Lockhart, Dr Kate Sidaway-Lee; Ide Lane Surgery: Dr John Campbell, Dr Sam Hilton, Sarah Manton, Dr Daniel Webber-Rookes, Rachel Winder; Travel Clinic: James Moore; Royal Devon and Exeter NHS Foundation Trust: Freya Bateman, Dr Michael Gibbons, Dr Bridget Knight, Julie Moss, Dr Sarah Statton, Josephine Studham; Teign Estuary Medical Group/Glendevon Medical Practice: Lydia Hall, Will Moyle, Dr Tamsin Venton.

Recruiting Centres (Site Leads)

Australia
Royal Children's Hospital Melbourne (**Prof Nigel Curtis**)
Monash Health (**Dr Tony Korman**)
Epworth HealthCare (**Dr Niki Tan)**
Fiona Stanley Hospital (**A/Prof Laurens Manning**)
Perth Children's Hospital (**Prof Peter Richmond**)
Sir Charles Gairdner Hospital (**Prof Michaela Lucas**)
Royal Adelaide Hospital (**Dr Simone Barry**)
Women's and Children's Hospital (**Prof Helen Marshall**)
St Vincent's Hospital, Sydney (**A/Prof Anthony Byrne**)
Prince of Wales Hospital (**Prof Jeffrey Post**)
Sydney Children's Hospital, Randwick (**Dr Brendan McMullan**)
The Children's Hospital at Westmead (**A/Prof Nicholas Wood**)
Westmead Hospital (**A/Prof Mark Douglas**)

Brazil
Federal University of Mato Grosso do Sul (**Prof Julio Croda**)
Hospital Regional de Mato Grosso do Sul (**Prof Julio Croda**)
CASSEMS Hospital (**Prof Julio Croda**)
Santa Casa Hospital (**Prof Julio Croda**)
Centro de Referência Prof Hélio Fraga (**Prof Margareth Dalcolmo**)
Centro de Estudos da Saúde do Trabalhador e Ecologia Humana (**Prof Margareth Dalcolmo**)
Fundação de Medicina Tropical Dr Heitor Vieira Dourado (**Dr Marcus Lacerda**)

The Netherlands
Noord West Ziekenhuis (**Dr Wim Boersma**)
Rijnstate Hospital (**Jet Gisolf)**
Amphia Hospital (**Prof Jan Kluytmans; Dr Wouter van den Bijllaardt**)
St Antonius Hospital (**Dr Bob Meek**)
Radboud UMC (**Dr Jaap ten Oever**)
UMC Utrecht (**Prof Marc Bonten**)

Spain
University Hospital German Trias I Pujol (**Dr Antoni Rosell**)
Mutua Terrassa University Hospital (**Tomás Perez Porcuna**)
University Hospital Cruces (**Dr Josune Goikoetxea**)
Marqués de Valdecilla University Hospital (**Dr María Carmen Fariñas Álvarez**)
University Hospital Virgen Macarena (**Prof Jesús Rodríguez-Baño**)

United Kingdom
St Leonard’s Practice (**Dr Alex Harding**)
Ide Lane Surgery (**Dr Daniel Webber-Rookes**)
Travel Clinic (**James Moore**)
Royal Devon and Exeter NHS Foundation Trust (**Dr Michael Gibbons**)
Teign Estuary Medical Group/Glendevon Medical Practice (**Dr Tamsin Venton**)

STATISTICAL ANALYSIS

The statistical analysis plan[11] was finalised and made publicly available before unblinding: all details, including the sample size calculation (n=7244), are available in the supplementary appendix. Assuming 4%/55% of participants would have severe/symptomatic COVID-19 by 6 months, and 16% loss to follow- up, we aimed to recruit 7,244 participants to provide 80% power to detect a 1.3% difference (2-sided alpha of 0.04) and 95% power to detect a 10% difference (2-sided alpha of 0.005) in the incidence of severe and symptomatic COVID-19, respectively.

Most analyses were done using a modified intention-to-treat (mITT) population, with participants analysed according to randomisation group, regardless of the intervention they received, restricted to participants who had a negative baseline SARS-CoV-2 test result. Selected sensitivity and supplementary analyses were done using the ITT population (without restriction). The primary outcomes were compared between the groups using a difference in probability of event by 6 months (presented as absolute difference in percentage).

Standardised survival curves were estimated for each arm, using flexible parametric (Royston-Parmar) models with adjustment for stratification factors. The 95% confidence intervals were estimated using bootstrapping. For the primary analysis, follow-up was censored at 6 months of follow-up, or at time of first COVID-19-specific vaccine, or when it could not be ascertained whether a COVID-19 episode had occurred (missing data for three consecutive days or more, or illness episode without COVID-19 test result).

Pre-planned supplementary analyses were done to provide additional insights: (i) including follow-up time after receipt of a COVID-19-specific vaccine; (ii) excluding episodes starting ≤14 days from randomisation; (iii) censoring participants at any subsequent vaccination (e.g., influenza vaccine); (iv) using the ITT population. Sensitivity analyses were also done: (i) restricted to episodes occurring after vaccination; (ii) using PCR/RAT results only (without serology) for defining COVID-19 episodes (in the ITT population); (iii) using less conservative censoring rules for missing data. See Table S1 for further details.

Pre-planned sub-group analyses were done by: (i) age group (<40 years/40 to 59 years/≥60 years); (ii) presence of comorbidities (yes/no, and by comorbidity); (iii) geographical location (Brazil/Europe); (iv) sex (male/female); (v) history of previous BCG (BCG-naïve/previous BCG) for the primary analysis.

Secondary outcomes were compared between arms with Cox regression models for time-to-event outcomes, logistic regression models for binary outcomes and zero-inflated negative binomial models for incidence rates. Proportional hazards assumptions were assessed using Schoenfeld residuals and the examination of log-log plots of survival (Figure S5). All models were adjusted for stratification factors.

Since the trial aimed to assess two primary outcomes, an adjustment for multiplicity was applied to maintain a global Type I error rate of 5% by splitting the alpha between the two primary outcomes (4% for severe COVID-19 and 1% for symptomatic COVID-19). An independent DSMB reviewed interim data at three meetings, including a single prespecified interim efficacy review of severe COVID-19 when 100 cases had been reached. A Pocock alpha-spending function was used to preserve overall alpha. A recommendation was given at the interim analysis to continue the trial.[14]

All the p-values are two-sided. No imputation was performed for missing outcomes, but the analysis method ensured all participants could be included in the analysis. No adjustment was made for testing multiple secondary outcomes; secondary efficacy outcomes were mostly components of the primary outcomes or closely related to it. Confidence interval widths have not been adjusted for multiplicity and may not be used in place of hypothesis testing. Full details are provided in the statistical analysis plan[11] in the supplementary appendix.

11. Orsini F. BRACE Statistical Analysis Plan for Interim Analysis. MCRI. 03/06/2021 (https://mcri.figshare.com/articles/online_resource/BRACE_Statistical_Analysis_Plan_for_Interim_Analysi s/14721309).

14. Orsini F. BRACE Trial SAP. MCRI. 29/08/2022 (https://mcri.figshare.com/articles/online_resource/BRACE_Trial_SAP/19836157).

FIGURES AND TABLES

Table S1. Overview of Primary, Sensitivity and Supplementary Estimands

Estimand	Brief Description of Estimand	Full Description of Estimand
1.1	Primary estimand<br>(Censoring participants at the time of COVID-19 vaccination)	To determine if BCG vaccination compared with placebo reduces the incidence of **symptomatic** COVID-19 in the absence of a COVID-19 specific vaccine, over the 6 months following randomisation, in healthcare workers who did not have a previous SARS-CoV-2 positive test result when assessed at time of randomisation.
1.2	Including follow-up time after COVID-19 vaccination	To determine if BCG vaccination compared with placebo reduces the incidence of **symptomatic** COVID-19 **irrespective of receiving a COVID-19-specific vaccine or any other vaccine**, over the 6 months following randomisation, in healthcare workers who did not have a previous SARS-CoV-2 positive test result when assessed at time of randomisation.
1.3	Excluding episode ≤14 days from randomisation	To determine if BCG vaccination compared with placebo reduces the incidence of **symptomatic** COVID-19 **following the 14 days after randomisation** in the absence of any COVID-19-specific vaccine, over the 6 months, in healthcare workers who did not have a previous SARS-CoV-2 positive test result when assessed at time of randomisation.
1.4	Censoring participants at the time of any vaccination	To determine if BCG vaccination compared with placebo reduces the incidence of **symptomatic** COVID-19 **in the absence of any vaccine (including COVID-19-specific vaccine)**, over the 6 months following randomisation, in healthcare workers who did not have a previous SARS-CoV-2 positive test result when assessed at time of randomisation.
1.5	Using the ITT population	To determine if BCG vaccination compared with placebo reduces the incidence of **symptomatic** COVID-19 in the absence of a COVID-19-specific vaccine, over the 6 months following randomisation, **in healthcare workers**
1.1_s1	Including episodes on or after trial vaccination date	To determine if BCG vaccination compared with placebo reduces the incidence of **symptomatic** COVID-19 in the absence of a COVID-19 specific vaccine, over the 6 months **following vaccination**, in healthcare workers who did not have a previous SARS-CoV-2 positive test result when assessed at time of randomisation.
1.1_s2	Using PCR/RAT results only to determine a COVID-19 episode (ITT)	To determine if BCG vaccination compared with placebo reduces the incidence of **symptomatic** COVID-19 (**determined using PCR/RAT tests only**) in the absence of a COVID-19 specific vaccine, over the 6 months following randomisation, in healthcare workers.
1.1_s3	Using less conservative censoring rules for missing data	Where data will be censored at the earlier of: [A] their first COVID-19 specific vaccine dose or [B] day 182 of their participation in the trial or [E] date of withdrawal/last contact unless the outcome is met and the first day with symptoms for their first symptomatic episode precedes [A], [B] and [E]. **An episode of illness with symptomatic COVID-19 symptoms where COVID-19 is unable to be excluded due to missing data will be ignored from the censoring algorithm (instead of censoring at this occurrence as in the primary analysis)**

2.1	Primary analysis<br>(Censoring participants at the time of COVID-19 vaccination)	To determine if BCG vaccination compared with placebo reduces the incidence of **severe** COVID-19 in the absence of a COVID-19 specific vaccine, over the 6 months following randomisation, in healthcare workers who did not have a previous SARS-CoV-2 positive test result when assessed at time of randomisation.
2.2	Including follow-up time after COVID-19 vaccination	To determine if BCG vaccination compared with placebo reduces the incidence of **severe** COVID-19 **irrespective of receiving a COVID-19-specific vaccine or any other vaccine**, over the 6 months following randomisation, in healthcare workers who did not have a previous SARS-CoV-2 positive test result when assessed at time of randomisation.
2.3	Excluding episode ≤14 days from randomisation	To determine if BCG vaccination compared with placebo reduces the incidence of **severe** COVID-19 f**ollowing the 14 days after randomisation** in the absence of any COVID-19-specific vaccine, over the 6 months, in healthcare workers who did not have a previous SARS-CoV-2 positive test result when assessed at time of randomisation.
2.4	Censoring participants at the time of any vaccination	To determine if BCG vaccination compared with placebo reduces the incidence of **severe** COVID-19 **in the absence of any vaccine (including COVID-19-specific vaccine)**, over the 6 months following randomisation, in healthcare workers who did not have a previous SARS-CoV-2 positive test result when assessed at time of randomisation.
2.5	Using the ITT population	To determine if BCG vaccination compared with placebo reduces the incidence of **severe** COVID-19 in the absence of a COVID-19-specific vaccine, over the 6 months following randomisation, **in healthcare workers.**
2.1_s1	Including episodes on or after trial vaccination date	To determine if BCG vaccination compared with placebo reduces the incidence of **severe** COVID-19 in the absence of a COVID-19 specific vaccine, over the 6 months **following vaccination**, in healthcare workers who did not have a previous SARS-CoV-2 positive test result when assessed at time of randomisation.
2.1_s2	Using PCR/RAT results only to determine a COVID-19 episode (ITT)	To determine if BCG vaccination compared with placebo reduces the incidence of **severe** COVID-19 (**determined using PCR/RAT tests only**) in the absence of a COVID-19 specific vaccine, over the 6 months following randomisation, in healthcare workers.
2.1_s3	Using less conservative censoring rules for missing data	Where data will be censored at the earlier of: [A] their first COVID-19 specific vaccine dose or[B] day 182 of their participation in the trial or [E] date of withdrawal/last contact unless the outcome is met and the first day with symptoms for their first severe episode precedes [A], [B] and [E]. **An episode of illness with severe COVID-19 symptoms where a COVID-19 episode is unable to be determined due to missing data will be ignored from the censoring algorithm (instead of censoring at this occurrence as in the primary analysis)**

Table S2. Additional Baseline Characteristics of mITT Population

	BCG	Placebo
**Participants included in mITT population**	**1703**	**1683**
BMI		
<18.5 kg/m^2	21/1672 (1.26%)	18/1638 (1.10%)
18.5 to 24.9 kg/m^2	667/1672 (39.89%)	667/1638 (40.72%)
25 to 29.9 kg/m^2	622/1672 (37.20%)	615/1638 (37.55%)
≥30 kg/m^2	362/1672 (21.65%)	338/1638 (20.63%)
WORKPLACE		
Emergency Department	77/1703 (4.52%)	89/1683 (5.29%)
Intensive Care Unit / High Dependency Unit	112/1703 (6.58%)	98/1683 (5.82%)
Operating Theatre	68/1703 (3.99%)	75/1683 (4.46%)
General ward	210/1703 (12.33%)	182/1683 (10.81%)
Pharmacy	46/1703 (2.70%)	52/1683 (3.09%)
Other ward/area	854/1703 (50.15%)	850/1683 (50.51%)
Paramedic / Ambulance	23/1703 (1.35%)	23/1683 (1.37%)
Aged care facility	29/1703 (1.70%)	29/1683 (1.72%)
Practice outside of hospital	284/1703 (16.68%)	285/1683 (16.93%)
PATIENT CONTACT WEEKLY		
No direct patient contact	340/1703 (19.96%)	342/1683 (20.32%)
<10 hours	266/1703 (15.62%)	275/1683 (16.34%)
10 - 20 hours	268/1703 (15.74%)	243/1683 (14.44%)
>20 hours	829/1703 (48.68%)	823/1683 (48.90%)
CONFIRMED COVID-19 PATIENT IN DEPARTMENT	1072/1703 (62.95%)	1044/1683 (62.03%)
SMOKING		
No	1527/1703 (89.67%)	1499/1683 (89.07%)
Yes, rarely (1 or 2 cigarettes a month)	29/1703 (1.70%)	39/1683 (2.32%)
Yes, occasionally (1 or 2 cigarettes a week)	39/1703 (2.29%)	30/1683 (1.78%)
Yes, regularly	108/1703 (6.34%)	115/1683 (6.83%)
BCG VACCINATION IN THE PAST		
No	441/1703 (25.90%)	439/1683 (26.08%)
Yes - 1 to 5 years ago	15/1703 (0.88%)	18/1683 (1.07%)
Yes - Greater than 5 years ago	1247/1703 (73.22%)	1226/1683 (72.85%)
BCG SCAR AT RANDOMISATION		
No	582/1701 (34.22%)	583/1683 (34.64%)
Yes	1093/1701 (64.26%)	1074/1683 (63.81%)
Unsure	26/1701 (1.53%)	26/1683 (1.54%)
TB EXPOSURE		
No	1686/1703 (99.00%)	1666/1683 (98.99%)
Yes	11/1703 (0.65%)	8/1683 (0.48%)
Not sure	6/1703 (0.35%)	9/1683 (0.53%)
POSITIVE TUBERCULIN SKIN TEST OR MANTOUX TEST		
No	1508/1703 (88.55%)	1464/1683 (86.99%)
Yes	94/1703 (5.52%)	114/1683 (6.77%)
Not sure	101/1703 (5.93%)	105/1683 (6.24%)

**SARS-COV-2 SEROLOGY AND RESPIRATORY SWAB AT BASELINE**		
PCR/SARS-COV-2 DIAGNOSTIC ANTIGEN TEST/SEROLOGY RESULTS		
All Negative	1703/1703 (100.00%)	1683/1683 (100.00%)
SEROLOGY RESULTS		
Negative	1703/1703 (100.00%)	1683/1683 (100.00%)
SWAB at randomisation (South America)		
Negative	1006/1006 (100.00%)	999/999 (100.00%)
**COMORBIDITIES**		
Presence of comorbidities (excluding BMI ≥30 kg/m^2)	356/1703 (20.90%)	333/1683 (19.79%)
Number of co-morbidities (1,2,3)		
1	317/356 (89.04%)	296/333 (88.89%)
2	38/356 (10.67%)	34/333 (10.21%)
3	1/356 (0.28%)	3/333 (0.90%)
Presence of comorbidities (including BMI ≥30 kg/m^2)	589/1672 (35.23%)	554/1638 (33.82%)
Diabetes	52/1703 (3.05%)	67/1683 (3.98%)
Type of diabetes		
Type 1 diabetes only	7/52 (13.46%)	8/67 (11.94%)
Type 2 diabetes only	43/52 (82.69%)	52/67 (77.61%)
Type 1 and type 2 diabetes	0/52 (0.00%)	1/67 (1.49%)
Unsure	2/52 (3.85%)	5/67 (7.46%)
Other type of diabetes	0/52 (0.00%)	1/67 (1.49%)
Chronic respiratory disease	111/1703 (6.52%)	92/1683 (5.47%)
Cardiovascular disease (any)	233/1703 (13.68%)	214/1683 (12.72%)
Ischaemic heart disease	5/233 (2.15%)	7/214 (3.27%)
Congestive heart disease	0/233 (0.00%)	2/214 (0.93%)
Other	18/233 (7.73%)	12/214 (5.61%)
Unsure	1/233 (0.43%)	1/214 (0.47%)
Hypertension	220/1703 (12.92%)	200/1683 (11.88%)
BMI≥30 kg/m^2	362/1672 (21.65%)	338/1638 (20.63%)
**OTHER SUBGROUPS**		
Subgroup 1 – By age group (<40 / 40-59 / 60+)		
<40 years old	740/1703 (43.45%)	734/1683 (43.61%)
40-59 years old	811/1703 (47.62%)	802/1683 (47.65%)
60+ years old	152/1703 (8.93%)	147/1683 (8.73%)
Subgroup 3 – By geographical location (Australia vs Europe vs South America)		
Australia	214/1703 (12.57%)	206/1683 (12.24%)
Europe	483/1703 (28.36%)	478/1683 (28.40%)
South America	1006/1703 (59.07%)	999/1683 (59.36%)
Subgroup 4 – By sex (female vs male)		
Female	1245/1700 (73.24%)	1281/1683 (76.11%)
Male	455/1700 (26.76%)	402/1683 (23.89%)
Subgroup 5 – By BCG in the past or not		
No BCG in the past (BCG naïve)	441/1703 (25.90%)	439/1683 (26.08%)
BCG in the past	1262/1703 (74.10%)	1244/1683 (73.92%)
Subgroup 6 – By serology results to SARS-CoV-2 when enrolling into the trial (negative vs non-negative)		
Negative serology at enrolment	1703/1703 (100.00%)	1683/1683 (100.00%)

Table S3 Additional Baseline Characteristics of ITT Population

	BCG	Placebo
**Participants included in ITT population**	**1999**	**1989**
BMI		
<18.5 kg/m^2	26/1967 (1.32%)	25/1941 (1.29%)
18.5 to 24.9 kg/m^2	752/1967 (38.23%)	773/1941 (39.82%)
25 to 29.9 kg/m^2	747/1967 (37.98%)	726/1941 (37.40%)
≥30 kg/m^2	442/1967 (22.47%)	417/1941 (21.48%)
WORKPLACE		
Emergency Department	100/1999 (5.00%)	120/1989 (6.03%)
Intensive Care Unit / High Dependency Unit	127/1999 (6.35%)	112/1989 (5.63%)
Operating Theatre	70/1999 (3.50%)	83/1989 (4.17%)
General ward	235/1999 (11.76%)	216/1989 (10.86%)
Pharmacy	73/1999 (3.65%)	77/1989 (3.87%)
Other ward / area	1014/1999 (50.73%)	1012/1989 (50.88%)
Paramedic / Ambulance	24/1999 (1.20%)	24/1989 (1.21%)
Aged care facility	31/1999 (1.55%)	33/1989 (1.66%)
Practice outside of hospital	325/1999 (16.26%)	312/1989 (15.69%)
PATIENT CONTACT WEEKLY		
No direct patient contact	377/1999 (18.86%)	372/1989 (18.70%)
<10 hours	337/1999 (16.86%)	361/1989 (18.15%)
10 - 20 hours	315/1999 (15.76%)	287/1989 (14.43%)
>20 hours	970/1999 (48.52%)	969/1989 (48.72%)
CONFIRMED COVID-19 PATIENT IN DEPARTMENT	1275/1999 (63.78%)	1265/1989 (63.60%)
SMOKING		
No	1803/1999 (90.20%)	1778/1989 (89.39%)
Yes, rarely (1 or 2 cigarettes a month)	37/1999 (1.85%)	46/1989 (2.31%)
Yes, occasionally (1 or 2 cigarettes a week)	42/1999 (2.10%)	38/1989 (1.91%)
Yes, regularly	117/1999 (5.85%)	127/1989 (6.39%)
BCG VACCINATION IN THE PAST		
No	462/1999 (23.11%)	468/1989 (23.53%)
Yes - 1 to 5 years ago	18/1999 (0.90%)	21/1989 (1.06%)
Yes - Greater than 5 years ago	1519/1999 (75.99%)	1500/1989 (75.41%)
BCG SCAR AT RANDOMISATION		
No	639/1996 (32.01%)	637/1988 (32.04%)
Yes	1330/1996 (66.63%)	1323/1988 (66.55%)
Unsure	27/1996 (1.35%)	28/1988 (1.41%)
TB EXPOSURE		
No	1981/1999 (99.10%)	1972/1989 (99.15%)
Yes	11/1999 (0.55%)	8/1989 (0.40%)
Not sure	7/1999 (0.35%)	9/1989 (0.45%)
POSITIVE TUBERCULIN SKIN TEST OR MANTOUX TEST		
No	1793/1999 (89.69%)	1760/1989 (88.49%)
Yes	101/1999 (5.05%)	122/1989 (6.13%)
Not sure	105/1999 (5.25%)	107/1989 (5.38%)

**SARS-COV-2 SEROLOGY AND SWAB AT BASELINE**		
PCR/SARS-COV-2 DIAGNOSTIC ANTIGEN TEST/SEROLOGY RESULTS		
All negative	1703/1999 (85.19%)	1683/1989 (84.62%)
At least one positive	290/1999 (14.51%)	300/1989 (15.08%)
Inconclusive / Not performed / Missing	6/1999 (0.30%)	6/1989 (0.30%)
SEROLOGY RESULTS		
Negative	1720/1999 (86.04%)	1699/1989 (85.42%)
Positive	275/1999 (13.76%)	286/1989 (14.38%)
Not done	2/1999 (0.10%)	2/1989 (0.10%)
Done but missing results	2/1999 (0.10%)	2/1989 (0.10%)
SWAB at randomisation (South America)		
Negative	1245/1285 (96.89%)	1241/1283 (96.73%)
Positive	34/1285 (2.65%)	36/1283 (2.81%)
Inconclusive	2/1285 (0.16%)	1/1283 (0.08%)
Not performed	4/1285 (0.31%)	5/1283 (0.39%)
**COMORBIDITIES**		
Presence of comorbidities (excluding BMI ≥30 kg/m^2)	400/1999 (20.01%)	389/1989 (19.56%)
Number of co-morbidities (1,2,3)		
1	352/400 (88.00%)	349/389 (89.72%)
2	47/400 (11.75%)	37/389 (9.51%)
3	1/400 (0.25%)	3/389 (0.77%)
Presence of comorbidities (ANY)	695/1967 (35.33%)	669/1941 (34.47%)
Diabetes	62/1999 (3.10%)	74/1989 (3.72%)
Type of diabetes		
Type 1 diabetes only	10/62 (16.13%)	8/74 (10.81%)
Type 2 diabetes only	49/62 (79.03%)	56/74 (75.68%)
Type 1 and type 2 diabetes	0/62 (0.00%)	1/74 (1.35%)
Unsure	3/62 (4.84%)	8/74 (10.81%)
Other type of diabetes	0/62 (0.00%)	1/74 (1.35%)
Chronic respiratory disease	126/1999 (6.30%)	108/1989 (5.43%)
Cardiovascular disease (any)	261/1999 (13.06%)	250/1989 (12.57%)
Ischaemic heart disease	5/261 (1.92%)	7/250 (2.80%)
Congestive heart disease	0/261 (0.00%)	2/250 (0.80%)
Other	19/261 (7.28%)	13/250 (5.20%)
Unsure	1/261 (0.38%)	1/250 (0.40%)
Hypertension	247/1999 (12.36%)	236/1989 (11.87%)
BMI ≥30 kg/m2	442/1967 (22.47%)	417/1941 (21.48%)

**OTHER SUBGROUPS**		
Subgroup 1 – By age group (<40 / 40-59 / 60+)		
<40 years old	916/1999 (45.82%)	914/1989 (45.95%)
40-59 years old	924/1999 (46.22%)	921/1989 (46.30%)
60+ years old	159/1999 (7.95%)	154/1989 (7.74%)
Subgroup 3 – By geographical location (Australia vs Europe vs South America)		
Australia	216/1999 (10.81%)	206/1989 (10.36%)
Europe	498/1999 (24.91%)	500/1989 (25.14%)
South America	1285/1999 (64.28%)	1283/1989 (64.50%)
Subgroup 4 – By sex (female vs male)		
Female	1446/1996 (72.44%)	1494/1989 (75.11%)
Male	550/1996 (27.56%)	495/1989 (24.89%)
Subgroup 5 – By BCG in the past or not		
No BCG in the past (BCG naïve)	462/1999 (23.11%)	468/1989 (23.53%)
BCG in the past	1537/1999 (76.89%)	1521/1989 (76.47%)
Subgroup 6 – By serology results to SARS-CoV-2 when enrolling into the trial		
Non-negative (i.e., positive / missing / indeterminate) serology at enrolment	296/1999 (14.81%)	306/1989 (15.38%)
Negative serology at enrolment	1703/1999 (85.19%)	1683/1989 (84.62%)

Table S4 Baseline Characteristics of Safety Population

	BCG (Received)	Placebo (Received)	No Intervention Received	Not sure which Intervention Received
**Participants included in Safety Population**	**1996**	**1982**	**9**	**1**
SEX Female	1443/1996 (72.29%)	1489/1982 (75.13%)	8/9 (88.89%)	0/1 (0.00%)
Age, Mean (SD)	1996, 42.0 (12.1)	1982, 42.0 (12.1)	9, 41.4 (14.0)	1, 53.2 (.)
COUNTRY				
Australia	211/1996 (10.57%)	208/1982 (10.49%)	3/9 (33.33%)	0/1 (0.00%)
Europe	500/1996 (25.05%)	496/1982 (25.03%)	1/9 (11.11%)	1/1 (100.00%)
South America	1285/1996 (64.38%)	1278/1982 (64.48%)	5/9 (55.56%)	0/1 (0.00%)
BMI				
<18.5 kg/m^2	26/1965 (1.32%)	25/1934 (1.29%)	0/8 (0.00%)	0/1 (0.00%)
18.5 to 24.9 kg/m^2	753/1965 (38.32%)	769/1934 (39.76%)	2/8 (25.00%)	1/1 (100.00%)
25 to 29.9 kg/m^2	745/1965 (37.91%)	725/1934 (37.49%)	3/8 (37.50%)	0/1 (0.00%)
≥30 kg/m^2	441/1965 (22.44%)	415/1934 (21.46%)	3/8 (37.50%)	0/1 (0.00%)
WORKPLACE				
Emergency Department	99/1996 (4.96%)	120/1982 (6.05%)	1/9 (11.11%)	0/1 (0.00%)
Intensive Care Unit / High Dependency Unit	128/1996 (6.41%)	110/1982 (5.55%)	1/9 (11.11%)	0/1 (0.00%)
Operating Theatre	70/1996 (3.51%)	83/1982 (4.19%)	0/9 (0.00%)	0/1 (0.00%)
General ward	234/1996 (11.72%)	216/1982 (10.90%)	1/9 (11.11%)	0/1 (0.00%)
Pharmacy	72/1996 (3.61%)	77/1982 (3.88%)	1/9 (11.11%)	0/1 (0.00%)
Other ward/area	1014/1996 (50.80%)	1008/1982 (50.86%)	4/9 (44.44%)	0/1 (0.00%)
Paramedic / Ambulance	23/1996 (1.15%)	24/1982 (1.21%)	0/9 (0.00%)	1/1 (100.00%)
Aged care facility	30/1996 (1.50%)	33/1982 (1.66%)	1/9 (11.11%)	0/1 (0.00%)
Practice outside of hospital	326/1996 (16.33%)	311/1982 (15.69%)	0/9 (0.00%)	0/1 (0.00%)
WORK ROLE				
Nurse/Midwife	397/1996 (19.89%)	369/1982 (18.62%)	2/9 (22.22%)	0/1 (0.00%)
Doctor	212/1996 (10.62%)	193/1982 (9.74%)	0/9 (0.00%)	0/1 (0.00%)
Pharmacist	37/1996 (1.85%)	37/1982 (1.87%)	1/9 (11.11%)	0/1 (0.00%)
Patient Service Assistant	180/1996 (9.02%)	165/1982 (8.32%)	0/9 (0.00%)	0/1 (0.00%)
Clerical/Administrative duties	305/1996 (15.28%)	303/1982 (15.29%)	2/9 (22.22%)	0/1 (0.00%)
Other role	87/1996 (4.36%)	95/1982 (4.79%)	2/9 (22.22%)	0/1 (0.00%)
Paramedic / Ambulance paramedic	27/1996 (1.35%)	30/1982 (1.51%)	0/9 (0.00%)	1/1 (100.00%)
Carer	20/1996 (1.00%)	21/1982 (1.06%)	1/9 (11.11%)	0/1 (0.00%)
Allied Health	382/1996 (19.14%)	393/1982 (19.83%)	1/9 (11.11%)	0/1 (0.00%)

PSA/hospital maintenance	147/1996 (7.36%)	148/1982 (7.47%)	0/9 (0.00%)	0/1 (0.00%)
Scientist (medical/research)	86/1996 (4.31%)	88/1982 (4.44%)	0/9 (0.00%)	0/1 (0.00%)
Community Health Agent	87/1996 (4.36%)	100/1982 (5.05%)	0/9 (0.00%)	0/1 (0.00%)
Dentist/dental therapy	29/1996 (1.45%)	40/1982 (2.02%)	0/9 (0.00%)	0/1 (0.00%)
PATIENT CONTACT WEEKLY				
No direct patient contact	378/1996 (18.94%)	370/1982 (18.67%)	1/9 (11.11%)	0/1 (0.00%)
<10 hours	337/1996 (16.88%)	360/1982 (18.16%)	1/9 (11.11%)	0/1 (0.00%)
10 - 20 hours	312/1996 (15.63%)	288/1982 (14.53%)	2/9 (22.22%)	0/1 (0.00%)
>20 hours	969/1996 (48.55%)	964/1982 (48.64%)	5/9 (55.56%)	1/1 (100.00%)
CONFIRMED COVID-19 PATIENT IN DEPARTMENT	1277/1996 (63.98%)	1258/1982 (63.47%)	4/9 (44.44%)	1/1 (100.00%)
SMOKING				
No	1800/1996 (90.18%)	1771/1982 (89.35%)	9/9 (100.00%)	1/1 (100.00%)
Yes, rarely (1 or 2 cigarettes a month)	37/1996 (1.85%)	46/1982 (2.32%)	0/9 (0.00%)	0/1 (0.00%)
Yes, occasionally (1 or 2 cigarettes a week)	42/1996 (2.10%)	38/1982 (1.92%)	0/9 (0.00%)	0/1 (0.00%)
Yes, regularly	117/1996 (5.86%)	127/1982 (6.41%)	0/9 (0.00%)	0/1 (0.00%)
BCG VACCINATION IN THE PAST				
No	460/1996 (23.05%)	465/1982 (23.46%)	4/9 (44.44%)	1/1 (100.00%)
Yes - 1 to 5 years ago	18/1996 (0.90%)	21/1982 (1.06%)	0/9 (0.00%)	0/1 (0.00%)
Yes - Greater than 5 years ago	1518/1996 (76.05%)	1496/1982 (75.48%)	5/9 (55.56%)	0/1 (0.00%)
BCG SCAR AT RANDOMISATION				
No	637/1995 (31.93%)	636/1982 (32.09%)	3/6 (50.00%)	0/1 (0.00%)
Yes	1331/1995 (66.72%)	1319/1982 (66.55%)	2/6 (33.33%)	1/1 (100.00%)
Unsure	27/1995 (1.35%)	27/1982 (1.36%)	1/6 (16.67%)	0/1 (0.00%)
TB EXPOSURE				
No	1978/1996 (99.10%)	1965/1982 (99.14%)	9/9 (100.00%)	1/1 (100.00%)
Yes	11/1996 (0.55%)	8/1982 (0.40%)	0/9 (0.00%)	0/1 (0.00%)
Not sure	7/1996 (0.35%)	9/1982 (0.45%)	0/9 (0.00%)	0/1 (0.00%)
POSITIVE TUBERCULIN SKIN TEST OR MANTOUX TEST				
No	1790/1996 (89.68%)	1754/1982 (88.50%)	8/9 (88.89%)	1/1 (100.00%)
Yes	101/1996 (5.06%)	122/1982 (6.16%)	0/9 (0.00%)	0/1 (0.00%)
Not sure	105/1996 (5.26%)	106/1982 (5.35%)	1/9 (11.11%)	0/1 (0.00%)

**SARS-COV-2 SEROLOGY AND SWAB AT BASELINE**				
PCR/SARS-COV-2 DIAGNOSTIC ANTIGEN TEST/SEROLOGY RESULTS				
All negative	1702/1996 (85.27%)	1679/1982 (84.71%)	4/9 (44.44%)	1/1 (100.00%)
At least one positive	290/1996 (14.53%)	300/1982 (15.14%)	0/9 (0.00%)	0/1 (0.00%)
Inconclusive / Not performed / Missing	4/1996 (0.20%)	3/1982 (0.15%)	5/9 (55.56%)	0/1 (0.00%)
SEROLOGY RESULTS				
Negative	1720/1996 (86.17%)	1694/1982 (85.47%)	4/9 (44.44%)	1/1 (100.00%)
Positive	275/1996 (13.78%)	286/1982 (14.43%)	0/9 (0.00%)	0/1 (0.00%)
Not done	0/1996 (0.00%)	1/1982 (0.05%)	3/9 (33.33%)	0/1 (0.00%)
Done but missing results	1/1996 (0.05%)	1/1982 (0.05%)	2/9 (22.22%)	0/1 (0.00%)
SWAB at randomisation (South America)				
Negative	1245/1285 (96.89%)	1239/1278 (96.95%)	2/5 (40.00%)	0/0 (.%)
Positive	34/1285 (2.65%)	36/1278 (2.82%)	0/5 (0.00%)	0/0 (.%)
Inconclusive	3/1285 (0.23%)	0/1278 (0.00%)	0/5 (0.00%)	0/0 (.%)
Not performed	3/1285 (0.23%)	3/1278 (0.23%)	3/5 (60.00%)	0/0 (.%)
**COMORBIDITIES**				
Presence of comorbidities (excluding BMI ≥30 kg/m^2)	399/1996 (19.99%)	386/1982 (19.48%)	4/9 (44.44%)	0/1 (0.00%)
Number of co-morbidities (1,2,3)				
1	351/399 (87.97%)	347/386 (89.90%)	3/4 (75.00%)	0/0 (.%)
2	47/399 (11.78%)	36/386 (9.33%)	1/4 (25.00%)	0/0 (.%)
3	1/399 (0.25%)	3/386 (0.78%)	0/4 (0.00%)	0/0 (.%)
Presence of comorbidities (including BMI ≥30 kg/m^2)	694/1965 (35.32%)	665/1934 (34.38%)	5/8 (62.50%)	0/1 (0.00%)
Diabetes	62/1996 (3.11%)	73/1982 (3.68%)	1/9 (11.11%)	0/1 (0.00%)
What type of diabetes?				
Type 1 diabetes only	10/62 (16.13%)	8/73 (10.96%)	0/1 (0.00%)	0/0 (.%)
Type 2 diabetes only	49/62 (79.03%)	55/73 (75.34%)	1/1 (100.00%)	0/0 (.%)
Type 1 and type 2 diabetes	0/62 (0.00%)	1/73 (1.37%)	0/1 (0.00%)	0/0 (.%)
Unsure	3/62 (4.84%)	8/73 (10.96%)	0/1 (0.00%)	0/0 (.%)
Other type of diabetes	0/62 (0.00%)	1/73 (1.37%)	0/1 (0.00%)	0/0 (.%)
Chronic respiratory disease	125/1996 (6.26%)	108/1982 (5.45%)	1/9 (11.11%)	0/1 (0.00%)
Cardiovascular disease (any)	261/1996 (13.08%)	247/1982 (12.46%)	3/9 (33.33%)	0/1 (0.00%)
Ischaemic heart disease	5/261 (1.92%)	7/247 (2.83%)	0/3 (0.00%)	0/0 (.%)
Congestive heart disease	0/261 (0.00%)	2/247 (0.81%)	0/3 (0.00%)	0/0 (.%)
Other	19/261 (7.28%)	13/247 (5.26%)	0/3 (0.00%)	0/0 (.%)
Unsure	1/261 (0.38%)	1/247 (0.40%)	0/3 (0.00%)	0/0 (.%)

Hypertension	247/1996 (12.37%)	233/1982 (11.76%)	3/9 (33.33%)	0/1 (0.00%)
BMI ≥30 kg/m^2	441/1965 (22.44%)	415/1934 (21.46%)	3/8 (37.50%)	0/1 (0.00%)
**OTHER SUBGROUPS**				
Subgroup 1 – By age group (<40 / 40-59 / 60+)				
<40 years old	914/1996 (45.79%)	910/1982 (45.91%)	6/9 (66.67%)	0/1 (0.00%)
40-59 years old	924/1996 (46.29%)	918/1982 (46.32%)	2/9 (22.22%)	1/1 (100.00%)
60+ years old	158/1996 (7.92%)	154/1982 (7.77%)	1/9 (11.11%)	0/1 (0.00%)
Subgroup 3 – By geographical Location (Australia vs Europe vs South America)				
Australia	211/1996 (10.57%)	208/1982 (10.49%)	3/9 (33.33%)	0/1 (0.00%)
Europe	500/1996 (25.05%)	496/1982 (25.03%)	1/9 (11.11%)	1/1 (100.00%)
South America	1285/1996 (64.38%)	1278/1982 (64.48%)	5/9 (55.56%)	0/1 (0.00%)
Subgroup 4 – By sex (female vs male)				
Female	1443/1993 (72.40%)	1489/1982 (75.13%)	8/9 (88.89%)	0/1 (0.00%)
Male	550/1993 (27.60%)	493/1982 (24.87%)	1/9 (11.11%)	1/1 (100.00%)
Subgroup 5 – By BCG in the past or not				
No BCG in the past	460/1996 (23.05%)	465/1982 (23.46%)	4/9 (44.44%)	1/1 (100.00%)
BCG in the past	1536/1996 (76.95%)	1517/1982 (76.54%)	5/9 (55.56%)	0/1 (0.00%)
Subgroup 6 – By serology results to SARS-CoV-2 when enrolling into the trial (ne				
Non-negative (i.e., positive / missing / indeterminate) serology at enrolment	294/1996 (14.73%)	303/1982 (15.29%)	5/9 (55.56%)	0/1 (0.00%)
Negative serology at enrolment	1702/1996 (85.27%)	1679/1982 (84.71%)	4/9 (44.44%)	1/1 (100.00%)

Table S5 Follow-up Characteristics of mITT Population

	BCG	Placebo
**Participants included in mITT population**	**1703**	**1683**
**INTERVENTION RECEIVED**		
Vaccination (any) given?		
No	3/1703 (0.18%)	1/1683 (0.06%)
Yes	1700/1703 (99.82%)	1682/1683 (99.94%)
Vaccination not given on same day of randomisation		
Vaccination given on same day of randomisation	1694/1700 (99.65%)	1670/1682 (99.29%)
Vaccination NOT given on same day of randomisation	6/1700 (0.35%)	12/1682 (0.71%)
Received opposite intervention (no reason specified)	1/1703 (0.06%)	7/1683 (0.42%)
Received opposite intervention due to informatic bug	3/1703 (0.18%)	0/1683 (0.00%)
Not possible to determine what the participant received	1/1703 (0.06%)	0/1683 (0.00%)
**FOLLOW-UP**		
Months of follow-up, Median (IQR)	12.7 (12.3-13.2)	12.7 (12.3-13.2)
Months of follow-up prior to COVID-19 vaccination, Median (IQR)	2.9 (1.7-6.2)	2.7 (1.6-6.0)
Followed for 6months or more	1671/1703 (98.12%)	1648/1683 (97.92%)
**WITHDRAWALS IN THE FIRST 6 MONTHS**		
Withdrawal from the study?		
No	1676/1703 (98.41%)	1657/1683 (98.46%)
Yes	27/1703 (1.59%)	26/1683 (1.54%)
Reason for withdrawal		
Disappointed regarding allocation following randomisation	1/27	1/26
Study has become a burden	6/27	8/26
Lost to follow-up	3/27	1/26
Serious concurrent medical condition	2/27	0/26
Relocation	3/27	4/26
Consent withdrawn	5/27	10/26
No reason given	1/27	1/26
Personal reason / change in personal life	1/27	1/26
Other	5/27	0/26

**COVID19 VACCINATION IN THE FIRST 6 MONTHS**		
Received COVID-19 Specific Vaccines	1226/1703 (71.99%)	1231/1683 (73.14%)
Number of COVID-19 vaccine doses received		
1	259/1226 (21.13%)	218/1231 (17.71%)
2	967/1226 (78.87%)	1013/1231 (82.29%)
Days between Randomisation and COVID-19 Specific Vaccines, N, Mean (SD)	1652, 118.0 (86.3)	1631, 113.3 (83.8)
Brand of the 1st dose		
AstraZeneca/Oxford (ChAdOx1, Covishield)	459/1226 (37.44%)	445/1231 (36.15%)
2nd dose same brand	278/459 (60.57%)	289/445 (64.94%)
only 1 dose received	181/459 (39.43%)	155/445 (34.83%)
Pfizer/BioNTech (BNT162b2, Comirnaty)	245/1226 (19.98%)	234/1231 (19.01%)
2nd dose same brand	194/245 (79.18%)	194/234 (82.91%)
only 1 dose received	50/245 (20.41%)	40/234 (17.09%)
Moderna (mRNA-1273)	24/1226 (1.96%)	32/1231 (2.60%)
2nd dose same brand	10/24 (41.67%)	16/32 (50.00%)
only 1 dose received	14/24 (58.33%)	16/32 (50.00%)
Sinovac (CoronaVac)	495/1226 (40.38%)	520/1231 (42.24%)
2nd dose same brand	485/495 (97.98%)	513/520 (98.65%)
only 1 dose received	10/495 (2.02%)	7/520 (1.35%)
Johnson & Johnson (Ad26.COV2.S)	3/1226 (0.24%)	0/1231 (0.00%)
2nd dose same brand	NA	NA
only 1 dose received	3/3 (100.00%)	0/0 (0.00%)
**OTHER VACCINATIONS IN THE FIRST 6 MONTHS**		
Received other vaccines	616/1703 (36.17%)	601/1683 (35.71%)
Days between randomisation and other vaccine, N, Mean (SD)	942, 171.7 (90.9)	952, 178.4 (91.4)
Vaccine type of the first dose of other vaccine received		
Diphtheria-tetanus vaccine	16/616 (2.60%)	6/601 (1.00%)
Diphtheria-tetanus-pertussis vaccine	9/616 (1.46%)	4/601 (0.67%)
Diphtheria-tetanus-pertussis-polio vaccine	1/616 (0.16%)	1/601 (0.17%)
Hepatitis B vaccine	8/616 (1.30%)	9/601 (1.50%)
Hepatitis A vaccine	2/616 (0.32%)	0/601 (0.00%)
Typhoid oral vaccine	0/616 (0.00%)	1/601 (0.17%)
Influenza vaccine	566/616 (91.88%)	568/601 (94.51%)
Papillomavirus vaccine	7/616 (1.14%)	4/601 (0.67%)
Meningococcal vaccine	0/616 (0.00%)	1/601 (0.17%)
Pneumococcal vaccine	0/616 (0.00%)	1/601 (0.17%)
Rabies vaccine	3/616 (0.49%)	1/601 (0.17%)
Measles-Mumps-Rubella vaccine	2/616 (0.32%)	4/601 (0.67%)
Zoster live-attenuated vaccine	1/616 (0.16%)	0/601 (0.00%)
Zoster non live vaccine	1/616 (0.16%)	1/601 (0.17%)
Number of other vaccine doses received		
1	600/616 (97.40%)	586/601 (97.50%)
2	16/616 (2.60%)	15/601 (2.50%)

Table S6 Follow Characteristics of ITT Population

	BCG	Placebo
**Participants included in ITT population**	**1999**	**1989**
**INTERVENTION RECEIVED**		
Vaccination given? (Phase 2)		
No	6/1999 (0.30%)	3/1989 (0.15%)
Yes	1993/1999 (99.70%)	1986/1989 (99.85%)
Vaccination not given on same day of randomisation		
Vaccination given on same day of randomisation	1987/1993 (99.70%)	1974/1986 (99.40%)
Vaccination NOT given on same day of randomisation	6/1993 (0.30%)	12/1986 (0.60%)
Received opposite intervention (no reason specified)	1/1999 (0.05%)	8/1989 (0.40%)
Received opposite intervention due to informatic bug	3/1999 (0.15%)	0/1989 (0.00%)
Not possible to determine what the participant received	1/1999 (0.05%)	0/1989 (0.00%)
**FOLLOW-UP**		
Months of follow-up, Median (IQR)	12.7 (12.3-13.2)	12.7 (12.3-13.2)
Months of follow-up prior to COVID-19 vaccination, Median (IQR)	2.8 (1.5-5.8)	2.7 (1.5-5.8)
Followed for 6 months or more	1958/1999 (97.95%)	1945/1989 (97.79%)
**WITHDRAWALS IN THE FIRST 6 MONTHS**		
Withdrawal from the study?		
No	1964/1999 (98.25%)	1954/1989 (98.24%)
Yes	35/1999 (1.75%)	35/1989 (1.76%)
Reason for withdrawal		
Disappointed regarding allocation following randomisation	2/35	1/35
Study has become a burden	7/35	10/35
Lost to follow-up	3/35	1/35
Serious concurrent medical condition	2/35	0/35
Relocation	3/35	4/35
Consent withdrawn	7/35	14/35
No reason given	3/35	3/35
Personal reason / change in personal life	1/35	1/35
Other	7/35	1/35

**COVID19 VACCINATION IN THE FIRST 6 MONTHS**		
Received COVID-19 Specific Vaccines	1490/1999 (74.54%)	1486/1989 (74.71%)
Number of COVID-19 vaccine doses received		
1	321/1490 (21.54%)	282/1486 (18.98%)
2	1169/1490 (78.46%)	1204/1486 (81.02%)
Days between Randomisation and COVID-19 Specific Vaccines, N, Mean (SD)	1939, 112.0 (84.5)	1921, 109.2 (82.4)
Brand of the 1st dose		
AstraZeneca/Oxford (ChAdOx1, Covishield)	548/1490 (36.78%)	524/1486 (35.26%)
2nd dose same brand	332/548 (60.58%)	331/524 (63.17%)
only 1 dose received	216/548 (39.42%)	192/524 (36.64%)
Pfizer/BioNTech (BNT162b2, Comirnaty)	274/1490 (18.39%)	264/1486 (17.77%)
2nd dose same brand	202/274 (73.72%)	204/264 (77.27%)
only 1 dose received	70/274 (25.55%)	60/264 (22.73%)
Moderna (mRNA-1273)	24/1490 (1.61%)	35/1486 (2.36%)
2nd dose same brand	10/24 (41.67%)	17/35 (48.57%)
only 1 dose received	14/24 (58.33%)	18/35 (51.43%)
Sinovac (CoronaVac)	641/1490 (43.02%)	663/1486 (44.62%)
2nd dose same brand	623/641 (97.19%)	651/663 (98.19%)
only 1 dose received	18/641 (2.81%)	12/663 (1.81%)
Johnson & Johnson (Ad26.COV2.S)	3/1490 (0.20%)	0/1486 (0.00%)
2nd dose same brand	NA	NA
only 1 dose received	3/3 (100.00%)	0/0 (.%)
**OTHER VACCINATIONS IN THE FIRST 6 MONTHS**		
Received other vaccines	740/1999 (37.02%)	734/1989 (36.90%)
Days between randomisation and other vaccine, N, Mean (SD)	1083, 167.2 (88.1)	1108, 172.6 (89.8)
Vaccine type of the first dose of vaccine received		
Diphtheria-tetanus vaccine	18/740 (2.43%)	8/734 (1.09%)
Diphtheria-tetanus-pertussis vaccine	11/740 (1.49%)	4/734 (0.54%)
Diphtheria-tetanus-pertussis-polio vaccine	1/740 (0.14%)	1/734 (0.14%)
Hepatitis B vaccine	11/740 (1.49%)	12/734 (1.63%)
Hepatitis A vaccine	2/740 (0.27%)	1/734 (0.14%)
Hepatitis A-hepatitis B vaccine	0/740 (0.00%)	1/734 (0.14%)
Typhoid oral vaccine	0/740 (0.00%)	1/734 (0.14%)
Influenza vaccine	682/740 (92.16%)	693/734 (94.41%)
Papillomavirus vaccine	7/740 (0.95%)	5/734 (0.68%)
Meningococcal vaccine	0/740 (0.00%)	1/734 (0.14%)
Pneumococcal vaccine	0/740 (0.00%)	1/734 (0.14%)
Rabies vaccine	3/740 (0.41%)	1/734 (0.14%)
Measles-Mumps-Rubella vaccine	3/740 (0.41%)	4/734 (0.54%)
Zoster live-attenuated vaccine	1/740 (0.14%)	0/734 (0.00%)
Zoster non live vaccine	1/740 (0.14%)	1/734 (0.14%)
Number of other vaccine doses received		
1	717/740 (96.89%)	717/734 (97.68%)
2	23/740 (3.11%)	17/734 (2.32%)

Table S7 Primary Outcome (Estimand 1.1) – Incidence of Symptomatic COVID-19 by 6 Months (mITT)

	BCG	Placebo	Difference (BCG-Placebo)
Participants in mITT population	1703	1683	
Symptomatic COVID-19 by 6 months	132/1703 (7.75%)	106/1683 (6.30%)	
Censoring			
Censored- COVID-19 vaccination <6 months	1102/1703 (64.71%)	1131/1683 (67.20%)	
Censored- 6 months w/ no event	414/1703 (24.31%)	385/1683 (22.88%)	
Censored- missing/indeterminant test (serology/PCR/RAT)	29/1703 (1.70%)	23/1683 (1.37%)	
Censored- incomplete data entry/drop-out from the study	26/1703 (1.53%)	38/1683 (2.26%)	
Person years	445	431	
Event rate (per 100 person years)	29.44 95% CI (24.81 ; 34.94)	24.37 95% CI (20.13 ; 29.50)	
Estimated probability of Symptomatic COVID-19 by 6 months			
- Unadjusted	0.119 95% CI (0.099 ; 0.139)	0.098 95% CI (0.079 ; 0.118)	0.021 95% CI (-0.008 ; 0.049)
- Adjusted for stratification factors	0.147 95% CI (0.120 ; 0.173)	0.123 95% CI (0.097 ; 0.148)	0.024 95% CI (-0.007 ; 0.055)
- Adjusted for stratification factors + a priori baseline covariates	0.148 95% CI (0.120 ; 0.176)	0.123 95% CI (0.098 ; 0.148)	0.025 95% CI (-0.006 ; 0.056)

BCG: 7 participant(s) censored on randomisation date and 1 participant(s) had event starting on randomisation date.
Placebo: 7 participant(s) censored on randomisation date and 1 participant(s) had event starting on randomisation date.
Full model adjusted for stratification factors used at randomisation and sex.

Estimand 1.1 = To determine if BCG vaccination compared with placebo reduces the incidence of symptomatic COVID-19 in the absence of a COVID-19 specific vaccine, over the 6 months following randomisation, in healthcare workers who did not have a previous SARS-CoV-2 positive test result when assessed at time of randomisation.

Figure S1 Subgroup Analysis of Estimand 1.1 – Incidence of Symptomatic COVID-19 (mITT) by 6 Months by Treatment Arm and Sex
Models adjusted for stratification factors used at randomisation. Confidence interval widths have not been adjusted for multiplicity and should not be used for hypothesis testing.

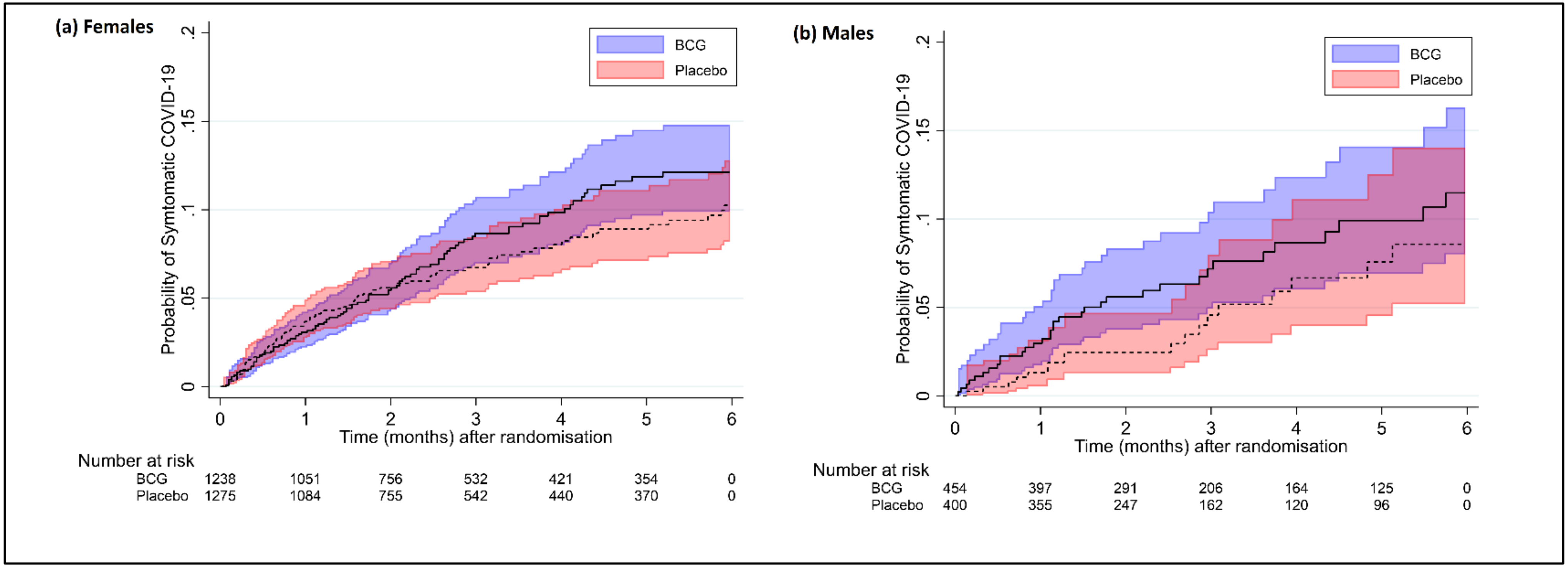

Table S8 Primary Outcome (Estimand 2.1) – Incidence of Severe COVID-19 by 6 Months (mITT)

	BCG	Placebo	Difference (BCG-Placebo)
Participants in mITT population	1703	1683	
Severe COVID-19 by 6 months	75/1703 (4.40%)	61/1683 (3.62%)	
Severe COVID-19 which resulted in death	0/75	1/61	
Severe COVID-19 which resulted in hospitalisation	5/75	4/61	
Non-hospitalised severe COVID-19	70/75	56/61	
Non-hospitalised severe COVID-19 too sick to work for ≥3 consecutive days	70/75	56/61	
Non-hospitalised severe COVID-19 confined to bed for ≥3 consecutive days	12/75	18/61	
Censoring			
Censored- COVID-19 vaccination <6 months	1171/1703 (68.76%)	1182/1683 (70.23%)	
Censored- 6 months w/ no event	427/1703 (25.07%)	398/1683 (23.65%)	
Censored- missing/indeterminant test (serology/PCR/RAT)	3/1703 (0.18%)	2/1683 (0.12%)	
Censored- incomplete data entry/drop-out from the study	27/1703 (1.59%)	40/1683 (2.38%)	
Person years	460	441	
Event rate (per 100 person years)	16.31<br>95% CI (13.00 ; 20.45)	13.83<br>95% CI (10.76 ; 17.77)	
Estimated probability of Symptomatic COVID-19 by 6 months			
- Unadjusted	0.067<br>95% CI (0.052 ; 0.083)	0.055<br>95% CI (0.040 ; 0.070)	0.012<br>95% CI (-0.010 ; 0.034)
- Adjusted for stratification factors	0.076<br>95% CI (0.058 ; 0.095)	0.065<br>95% CI (0.047 ; 0.082)	0.011<br>95% CI (-0.012 ; 0.035)
- Adjusted for stratification factors + a priori baseline covariates	0.077<br>95% CI (0.058 ; 0.097)	0.065<br>95% CI (0.047 ; 0.083)	0.013<br>95% CI (-0.010 ; 0.036)

BCG: 5 participant(s) censored on randomisation date and 0 participant(s) had event starting on randomisation date. Placebo: 7 participant(s) censored on randomisation date and 0 participant(s) had event starting on randomisation date. Full model adjusted for stratification factors used at randomisation and sex.

Estimand 2.1 = To determine if BCG vaccination compared with placebo reduces the incidence of severe COVID-19 in the absence of a COVID-19 specific vaccine, over the 6 months following randomisation, in healthcare workers who did not have a previous SARS-CoV-2 positive test result when assessed at time of randomisation.

Figure S2 Subgroup Analysis of Estimand 2.1 – Incidence of Severe COVID-19 (mITT) by 6 Months by Treatment Arm and Sex
Models adjusted for stratification factors used at randomisation. Confidence interval widths have not been adjusted for multiplicity and should not be used for hypothesis testing.

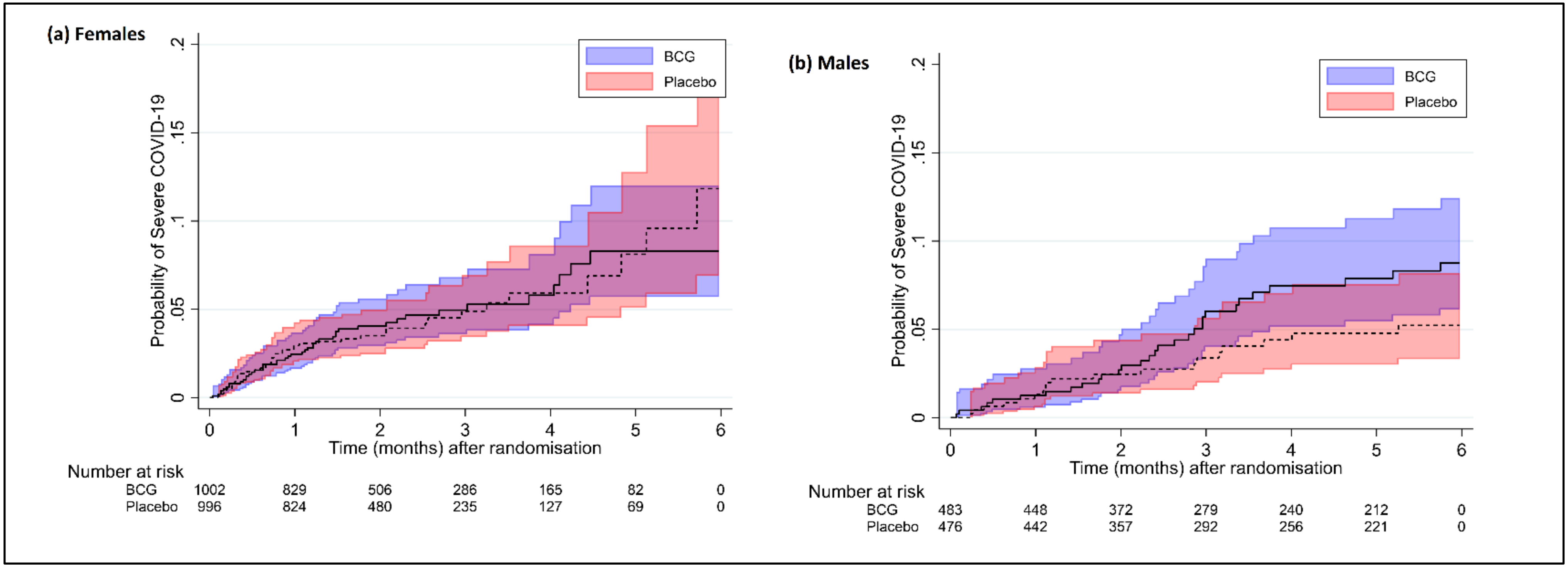

Table S9 Secondary Outcome (Estimand 5a.1) – Time to first COVID-19 Episode (Symptomatic or Severe) by 6 Months (mITT)

	BCG	Placebo	Hazard Ratio (BCG/Placebo)
Participants in mITT population	1703	1683	
COVID-19	135/1703 (7.93%)	107/1683 (6.36%)	
Symptomatic COVID-19	132/135	106/107	
Severe COVID-19	75/135	61/107	
Symptomatic COVID-19 (not severe)	60	46	
Severe COVID-19 (not symptomatic)	3	1	
Symptomatic Severe COVID-19	72	60	
Censoring			
Censored- COVID-19 vaccination <6 months	1098/1703 (64.47%)	1130/1683 (67.14%)	
Censored- 6 months w/ no event	414/1703 (24.31%)	385/1683 (22.88%)	
Censored- missing/indeterminant test (serology/PCR/RAT)	30/1703 (1.76%)	23/1683 (1.37%)	
Censored- incomplete data entry/drop-out from the study	26/1703 (1.53%)	38/1683 (2.26%)	
Person years	444	431	
Event rate (per 100 person years)	30.15 95% CI (25.46 ; 35.71)	24.62 95% CI (20.35 ; 29.78)	
Comparison of Time to First COVID-19 Episode by 6 Months			
- Unadjusted			1.23 95% CI (0.95; 1.59)
- Adjusted for stratification factors			1.23 95% CI (0.96; 1.59)
- Adjusted for stratification factors + a priori baseline covariates			1.21 95% CI (0.94; 1.56)

BCG: 7 participant(s) censored on randomisation date and 1 participant(s) had event starting on randomisation date.
Placebo: 7 participant(s) censored on randomisation date and 1 participant(s) had event starting on randomisation date.
Full model adjusted for stratification factors used at randomisation and sex, BMI ≥30 kg/m^2 and receipt of BCG in the past.

Estimand 5a.1 = To determine if BCG vaccination compared with placebo prolongs the time to first SARS-CoV-2-proven respiratory illness in the absence of a COVID-19 specific vaccine, measured over 6 months following randomisation in healthcare workers who did not have a previous SARS-CoV-2 positive test result when assessed at time of randomisation. Confidence interval widths have not been adjusted for multiplicity and should not be used for hypothesis testing.

Figure S3 Secondary Outcome (Estimand 5a.1) – Time to first COVID-19 Episode (Symptomatic or Severe) by 6 Months (mITT) – Primary Analysis, Sensitivity and Supplementary Analyses

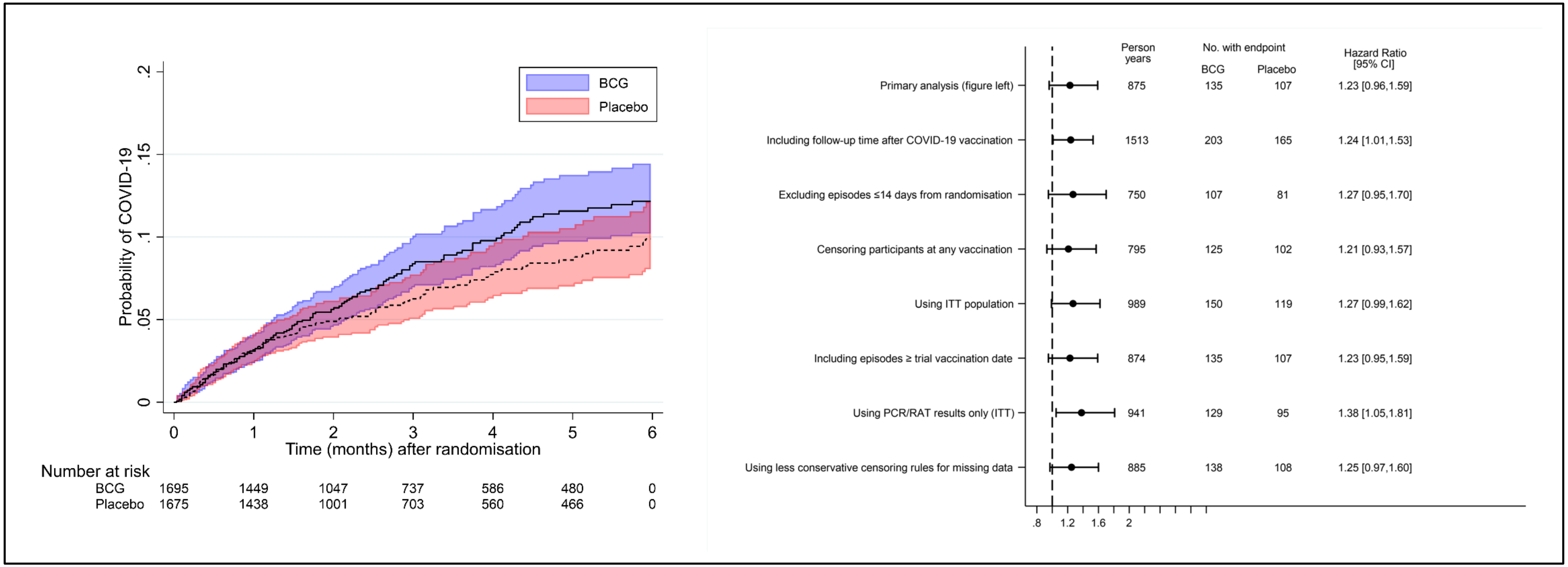

Primary Analysis – Estimand 5a.1 = To determine if BCG vaccination compared with placebo prolongs the time to first SARS-CoV-2-proven respiratory illness in the absence of a COVID-19 specific vaccine, measured over 6 months following randomisation in healthcare workers who did not have a previous SARS-CoV-2 positive test result when assessed at time of randomisation. Confidence interval widths have not been adjusted for multiplicity and should not be used for hypothesis testing.

Table S10 Secondary Outcome (Estimand 7a.1) – Asymptomatic SARS-CoV-2 Infection by 6 Months (mITT)

	BCG	Placebo	Difference (BCG-Placebo)
Participants in mITT population	1703	1683	
Participants who did not receive CoronaVac	1208/1703 (70.9%)	1163/1683 (69.1%)	
Participants with data	1071/1208 (88.7%)	978/1163 (84.1%)	
Asymptomatic COVID-19	12/1071 (1.12%)	15/978 (1.53%)	
Not asymptomatic COVID-19 as symptomatic/severe COVID-19	85/1059 (8.0%)	67/963 (7.0%)	
Not asymptomatic COVID-19 as no evidence of seroconversion	974/1059 (92.0%)	896/963 (93.0%)	
Estimated Proportion of Asymptomatic COVID-19 by 6 months			
- Unadjusted	0.0112 95% CI (0.0049 ; 0.0175)	0.0153 95% CI (0.0076 ; 0.0230)	-0.0041 95% CI (-0.0141 ; 0.0058)
- Adjusted for stratification factors	0.0113 95% CI (0.0049 ; 0.0176)	0.0153 95% CI (0.0076 ; 0.0229)	-0.0040 95% CI (-0.0139 ; 0.0059)
- Adjusted for stratification factors + a priori baseline covariates	0.0115 95% CI (0.0051 ; 0.0180)	0.0158 95% CI (0.0079 ; 0.0237)	-0.0043 95% CI (-0.0145 ; 0.0059)
- Reweighted + adjusted for stratification factors	0.0124 95% CI (0.0054 ; 0.0194)	0.0174 95% CI (0.0082 ; 0.0265)	-0.0050 95% CI (-0.0164 ; 0.0065)

Full model adjusted for stratification factors used at randomisation and sex, BMI ≥30 kg/m^2 and receipt of BCG in the past.

Estimand 7a.1 = To determine if BCG vaccination compared with placebo reduces the incidence of asymptomatic COVID-19 irrespective of receiving a non-CoronaVac COVID-19 specific vaccine or any other vaccine, over the 6 months following randomisation, in healthcare workers who did not receive CoronaVac during the study or have a previous SARS-CoV-2 positive test result when assessed at time of randomisation. Confidence interval widths have not been adjusted for multiplicity and should not be used for hypothesis testing.

Table S11 Baseline Characteristics of Participants with an Asymptomatic SARS-CoV-2 Infection by 6 Months (Estimand 7a.1)

	BCG	Placebo
Sex Female	10/12 (83.33%)	8/15 (53.33%)
Age, Mean (SD)	41.6 (13.0)	41.9 (12.2)
Country		
Europe	4/12 (33.33%)	2/15 (13.33%)
South America	8/12 (66.67%)	13/15 (86.67%)
BMI		
<18.5 kg/m^2	1/12 (8.33%)	1/15 (6.67%)
18.5 to 24.9 kg/m^2	5/12 (41.67%)	7/15 (46.67%)
25 to 29.9 kg/m^2	4/12 (33.33%)	5/15 (33.33%)
≥30 kg/^{m2}	2/12 (16.67%)	2/15 (13.33%)
Work Role		
Nurse / Midwife	2/12 (16.67%)	2/15 (13.33%)
Doctor	0/12 (0.00%)	2/15 (13.33%)
Patient Service Assistant	1/12 (8.33%)	1/15 (6.67%)
Clerical / Administrative duties	3/12 (25.00%)	0/15 (0.00%)
Other role	1/12 (8.33%)	2/15 (13.33%)
Allied Health	2/12 (16.67%)	3/15 (20.00%)
PSA / hospital maintenance	2/12 (16.67%)	5/15 (33.33%)
Scientist (medical/research)	1/12 (8.33%)	0/15 (0.00%)
Patient Contact Weekly		
No direct patient contact	4/12 (33.33%)	3/15 (20.00%)
<10 hours	0/12 (0.00%)	3/15 (20.00%)
>20 hours	8/12 (66.67%)	9/15 (60.00%)
Confirmed COVID-19 Patient in Department	9/12 (75.00%)	9/15 (60.00%)
Smoking		
No	11/12 (91.67%)	15/15 (100.00%)
Yes, rarely (1 or 2 cigarettes a month)	1/12 (8.33%)	0/15 (0.00%)
BCG Vaccination in the Past		
No	2/12 (16.67%)	1/15 (6.67%)
Yes - 1 to 5 years ago	0/12 (0.00%)	1/15 (6.67%)
Yes - Greater than 5 years ago	10/12 (83.33%)	13/15 (86.67%)

Table S12 Secondary Outcome (Estimand 6a.1) - Number of COVID-19 Episodes by 6 Months (mITT)

	BCG	Placebo	Difference in Logs of Expected Counts (BCG-Placebo)	Incidence Rate Ratio (BCG v Placebo)
Participants in mITT population	1703	1683		
Number of COVID-19 Episodes by 6 Months				
0	1568/1703 (92.1%)	1576/1683 (93.6%)		
1	133/1703 (7.8%)	103/1683 (6.1%)		
2	2/1703 (0.1%)	3/1683 (0.2%)		
3	0/1703 (0.0%)	1/1683 (0.1%)		
Number of COVID-19 Episodes by 6 Months in subgroup with COVID-19				
1	133/135 (98.5%)	103/107 (96.3%)		
2	2/135 (1.5%)	3/107 (2.8%)		
3	0/135 (0.0%)	1/107 (0.9%)		
Number of COVID-19 Episodes by 6 Months in subgroup with symptomatic COVID-19				
1	130/132 (98.5%)	102/106 (96.2%)		
2	2/132 (1.5%)	3/106 (2.8%)		
3	0/132 (0.0%)	1/106 (0.9%)		
Number of COVID-19 Episodes by 6 Months in subgroup with severe COVID-19				
1	73/75 (97.3%)	59/61 (96.7%)		
2	2/75 (2.7%)	1/61 (1.6%)		
3	0/75 (0.0%)	1/61 (1.6%)		
Comparison of Counts				
-Unadjusted			-0.047 95% CI (-0.297 ; 0.203)	0.954 95% CI (0.743 ; 1.225)
- Adjusted for stratification factors			-0.052 95% CI (-0.304 ; 0.199)	0.949 95% CI (0.738 ; 1.220)

Estimand 6a.1 = To determine if BCG vaccination compared with placebo reduces the number of COVID-19 episodes in the absence of a COVID-19 specific vaccine, measured over 6 months following randomisation in healthcare workers who did not have a previous SARS-CoV-2 positive test result when assessed at time of randomisation. Confidence interval widths have not been adjusted for multiplicity and should not be used for hypothesis testing.

Table S13 Secondary Outcome (Estimand 8a.1) - Number of Days Unable to Work due to COVID-19 over 6 Months (mITT)

	BCG	Placebo	Difference in Logs of Expected Counts (BCG-Placebo)	Incidence Rate Ratio (BCG v Placebo)
Participants in mITT population	1703	1683		
Number of Days Unable to Work, Median (IQR)	0.0 (0.0-0.0)	0.0 (0.0-0.0)		
Number of Days Unable to Work, Mean (SD)	0.7 (7.3)	0.4 (2.4)		
Number of Days Unable to Work in subgroup with COVID-19, Median (IQR)	3.0 (0.0-8.0)	4.0 (0.0-11.0)		
Number of Days Unable to Work in subgroup with COVID-19, Mean (SD)	8.6 (24.6)	6.4 (7.0)		
Number of Days Unable to Work in subgroup with symptomatic COVID-19, Median (IQR)	3.0 (0.0-8.5)	4.0 (0.0-11.0)		
Number of Days Unable to Work in subgroup with symptomatic COVID-19, Mean (SD)	8.7 (24.9)	6.4 (7.1)		
Number of Days Unable to Work in subgroup with severe COVID-19, Median (IQR)	8.0 (5.0-12.0)	9.0 (5.0-14.0)		
Number of Days Unable to Work in subgroup with severe COVID-19, Mean (SD)	15.0 (31.7)	10.6 (6.6)		
Comparison of Counts				
- Unadjusted			0.06<br>95% CI (-0.31; 0.42)	1.06<br>95% CI (0.73; 1.52)
- Adjusted for stratification factors			-0.13<br>95% CI (-0.49; 0.23)	0.88<br>95% CI (0.61; 1.26)

BCG: 7 participant(s) censored on randomisation date.
Placebo: 7 participant(s) censored on randomisation date.

Estimand 8a.1 = To determine if BCG vaccination compared with placebo reduces the number of days unable to work due to COVID-19 in the absence of a COVID-19 specific vaccine, measured over 6 months following randomisation in healthcare workers who did not have a previous SARS-CoV-2 positive test result when assessed at time of randomisation. Confidence interval widths have not been adjusted for multiplicity and should not be used for hypothesis testing.

Table S14 Secondary Outcome (Estimand 9a.1) - Number of Days Confined to Bed due to COVID-19 over 6 Months (mITT)

	BCG	Placebo	Difference in Logs of Expected Counts (BCG-Placebo)	Incidence Rate Ratio (BCG v Placebo)
Participants in mITT population	1703	1683		
Number of Days Confined to Bed, Median (IQR)	0.0 (0.0-0.0)	0.0 (0.0-0.0)		
Number of Days Confined to Bed, Mean (SD)	0.2 (3.0)	0.1 (1.1)		
Number of Days Confined to Bed in subgroup with COVID-19, Median (IQR)	0.0 (0.0-1.0)	0.0 (0.0-3.0)		
Number of Days Confined to Bed in subgroup with COVID-19, Mean (SD)	2.1 (10.3)	2.1 (3.8)		
Number of Days Confined to Bed in subgroup with symptomatic COVID-19, Median (IQR)	0.0 (0.0-1.0)	0.0 (0.0-3.0)		
Number of Days Confined to Bed in subgroup with symptomatic COVID-19, Mean (SD)	2.2 (10.4)	2.1 (3.8)		
Number of Days Confined to Bed in subgroup with severe COVID-19, Median (IQR)	1.0 (0.0-2.0)	2.0 (0.0-6.0)		
Number of Days Confined to Bed in subgroup with severe COVID-19, Mean (SD)	3.7 (13.7)	3.6 (4.5)		
Comparison of Counts				
- Unadjusted			-0.20<br>95% CI (-0.87 ; 0.46)	0.82<br>95% CI (0.42; 1.58)
- Adjusted for stratification factors			-0.28<br>95% CI (-0.96 ; 0.41)	0.76<br>95% CI (0.38; 1.50)

BCG: 7 participant(s) censored on randomisation date.
Placebo: 7 participant(s) censored on randomisation date.

Estimand 9a.1 = To determine if BCG vaccination compared with placebo reduces the number of days confined to bed due to COVID-19 in the absence of a COVID-19 specific vaccine, measured over 6 months following randomisation in healthcare workers who did not have a previous SARS-CoV-2 positive test result when assessed at time of randomisation. Confidence interval widths have not been adjusted for multiplicity and should not be used for hypothesis testing.

Figure S4 Covariate-adjusted Estimate of the Marginal Predicted Number of Days with Symptoms due to COVID-19 over 6 Months (mITT)

Models adjusted for stratification factors used at randomisation. Confidence interval widths have not been adjusted for multiplicity and should not be used for hypothesis testing.

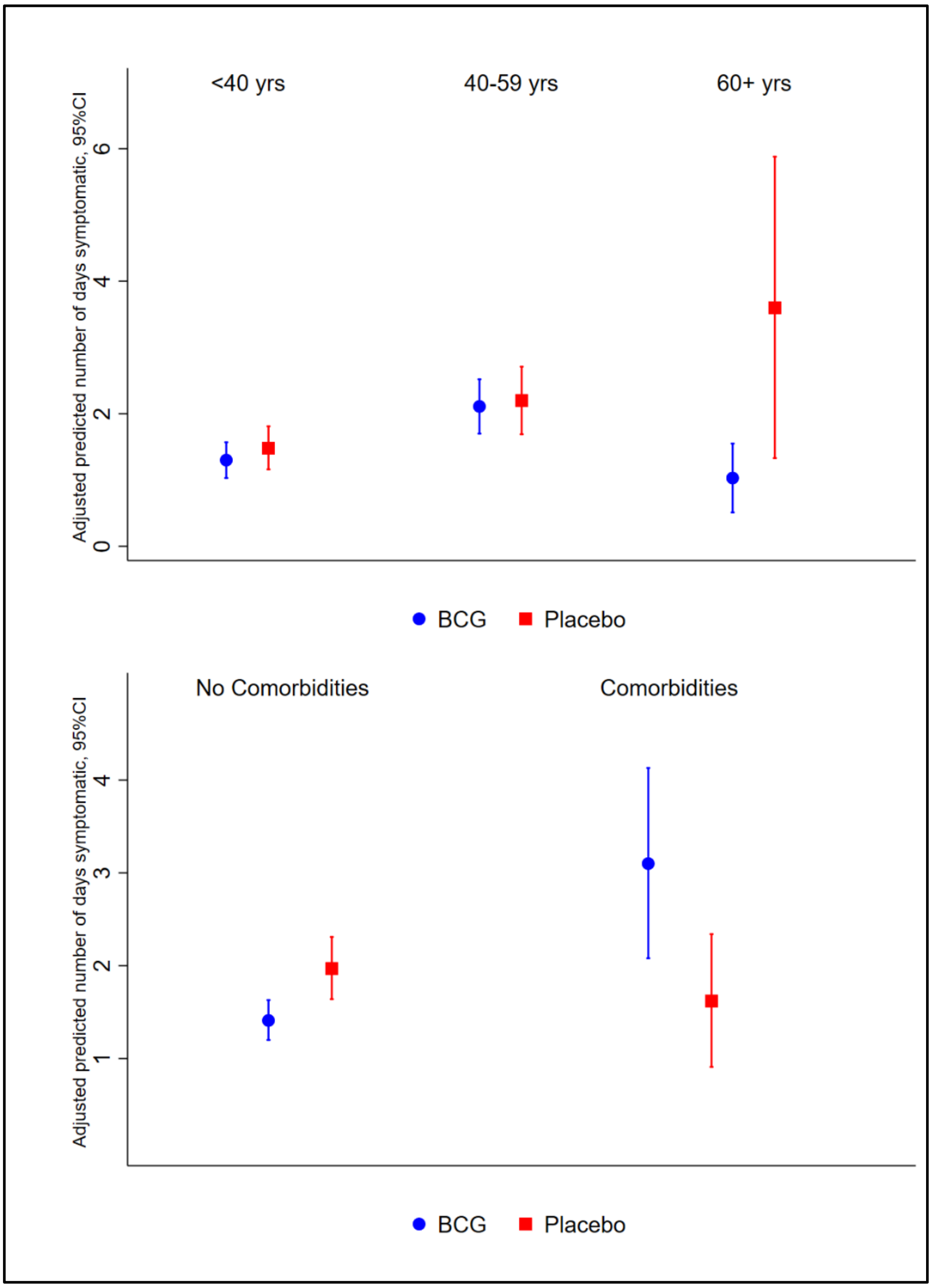

Table S15 Secondary Outcome (Estimand 11a.1) – Incidence of Pneumonia due to COVID-19 over 6 Months (mITT)

	BCG	Placebo	Hazard Ratio (BCG/Placebo)
Participants in mITT population	1703	1683	
Pneumonia due to COVID-19	7/1703 (0.41%)	7/1683 (0.42%)	
Censoring			
Censored- 6 months w/ no event	445/1703 (26.24%)	413/1683 (24.64%)	
Censored- COVID-19 vaccination <6 months	1190/1703 (70.17%)	1198/1683 (71.48%)	
Censored- incomplete data entry/drop-out from the study	28/1703 (1.65%)	38/1683 (2.27%)	
Censored- missing/indeterminant test (serology/PCR/RAT)	33/1703 (1.95%)	27/1683 (1.61%)	
Person years	471	452	
Event rate (per 100 person years)	1.49 95% CI (0.71 ; 3.12)	1.55 95% CI (0.74 ; 3.25)	
Comparison of Time to First Pneumonia due to COVID-19 Episode by 6 Months			
- Unadjusted			0.94 95% CI (0.33; 2.68)
- Adjusted for stratification factors			0.84 95% CI (0.29; 2.41)

BCG: 7 participant(s) censored on randomisation date and 0 participant(s) had event starting on randomisation date.
Placebo: 7 participant(s) censored on randomisation date and 0 participant(s) had event starting on randomisation date.

Estimand 11a.1 = To determine if BCG vaccination compared with placebo reduces the incidence of pneumonia due to COVID-19 in the absence of a COVID vaccine, measured over 6 months following randomisation in healthcare workers who did not have a previous SARS-CoV-2 positive test result when assessed at time of randomisation. Confidence interval widths have not been adjusted for multiplicity and should not be used for hypothesis testing.

Table S16 Secondary Outcome (Estimand 15a.1) – Incidence of Hospitalisation due to COVID-19 over 6 Months (mITT)

	BCG	Placebo	Hazard Ratio (BCG/Placebo)
Participants in mITT population	1703	1683	
Hospitalisation due to COVID-19	5/1703 (0.29%)	5/1683 (0.30%)	
Duration of Hospitalisation due to COVID-19 in Subgroup with Event, Median (IQR)	13.0 (5.0-18.0)	5.0 (3.0-7.0)	
Censoring			
Censored- 6 months w/ no event	445/1703 (26.21%)	413/1683 (24.61%)	
Censored- COVID-19 vaccination <6 months	1192/1703 (70.20%)	1200/1683 (71.51%)	
Censored- incomplete data entry/drop-out from the study	28/1703 (1.65%)	38/1683 (2.26%)	
Censored- missing/indeterminant test (serology/PCR/RAT)	33/1703 (1.94%)	27/1683 (1.61%)	
Person years	471	452	
Event rate (per 100 person years)	1.06 95% CI (0.44; 2.55)	1.11 95% CI (0.46; 2.66)	
Comparison of Time to First Hospitalisation due to COVID-19 Episode by 6 Months			
- Unadjusted			0.94 95% CI (0.27; 3.23)
- Adjusted for stratification factors			0.82 95% CI (0.23; 2.87)

BCG: 7 participant(s) censored on randomisation date and 0 participant(s) had event starting on randomisation date.
Placebo: 7 participant(s) censored on randomisation date and 0 participant(s) had event starting on randomisation date.

Estimand 15a.1 = To determine if BCG vaccination compared with placebo reduces the incidence of hospitalisation due to COVID-19 in the absence of a COVID vaccine, measured over 6 months following randomisation in healthcare workers who did not have a previous SARS-CoV-2 positive test result when assessed at time of randomisation. Confidence interval widths have not been adjusted for multiplicity and should not be used for hypothesis testing.

Table S17 Secondary Outcome (Estimand 29a.1) - Number of Days of Unplanned Absenteeism for Acute Illness or Hospitalisation over 6 Months (mITT)

	BCG	Placebo	Difference in Logs of Expected Counts (BCG-Placebo)	Incidence Rate Ratio (BCG v Placebo)
Participants in mITT population	1703	1683		
Participants with data	1634/1703 (95.9%)	1599/1683 (95.0%)		
Number of Days of Unplanned Absenteeism, Median (IQR)	0.0 (0.0-1.0)	0.0 (0.0-0.0)		
Number of Days of Unplanned Absenteeism, Mean (SD)	2.3 (7.1)	1.7 (4.9)		
Number of Days of Unplanned Absenteeism in subgroup with ≥1 day of absenteeism, Median (IQR)	6.0 (3.0-11.0)	6.0 (2.0-11.0)		
Number of Days of Unplanned Absenteeism in subgroup with ≥1 day of absenteeism, Mean (SD)	9.1 (11.7)	8.0 (7.8)		
Comparison of Counts				
-Unadjusted			0.13 95% CI (-0.004 ; 0.26)	1.14 95% CI (1.00 ; 1.29)
- Adjusted for stratification factors			0.11 95% CI (-0.01 ; 0.24)	1.12 95% CI (0.99 ; 1.27)

Estimand 29a.1 To determine if BCG vaccination compared with placebo reduces absenteeism, measured over 6 months following randomisation in healthcare workers who did not have a previous SARS-CoV-2 positive test result when assessed at time of randomisation. Confidence interval widths have not been adjusted for multiplicity and should not be used for hypothesis testing.

Figure S5 **Log(-log(survival)) Curves as a Function of Time (log scale) for the Primary Outcomes, Incidence of Severe and Symptomatic COVID-19 by 6 Months**

Models adjusted for stratification factors used at randomisation. Schoenfeld residuals p-values were p=0.816 for Symptomatic COVID and p=0.606 for Severe COVID

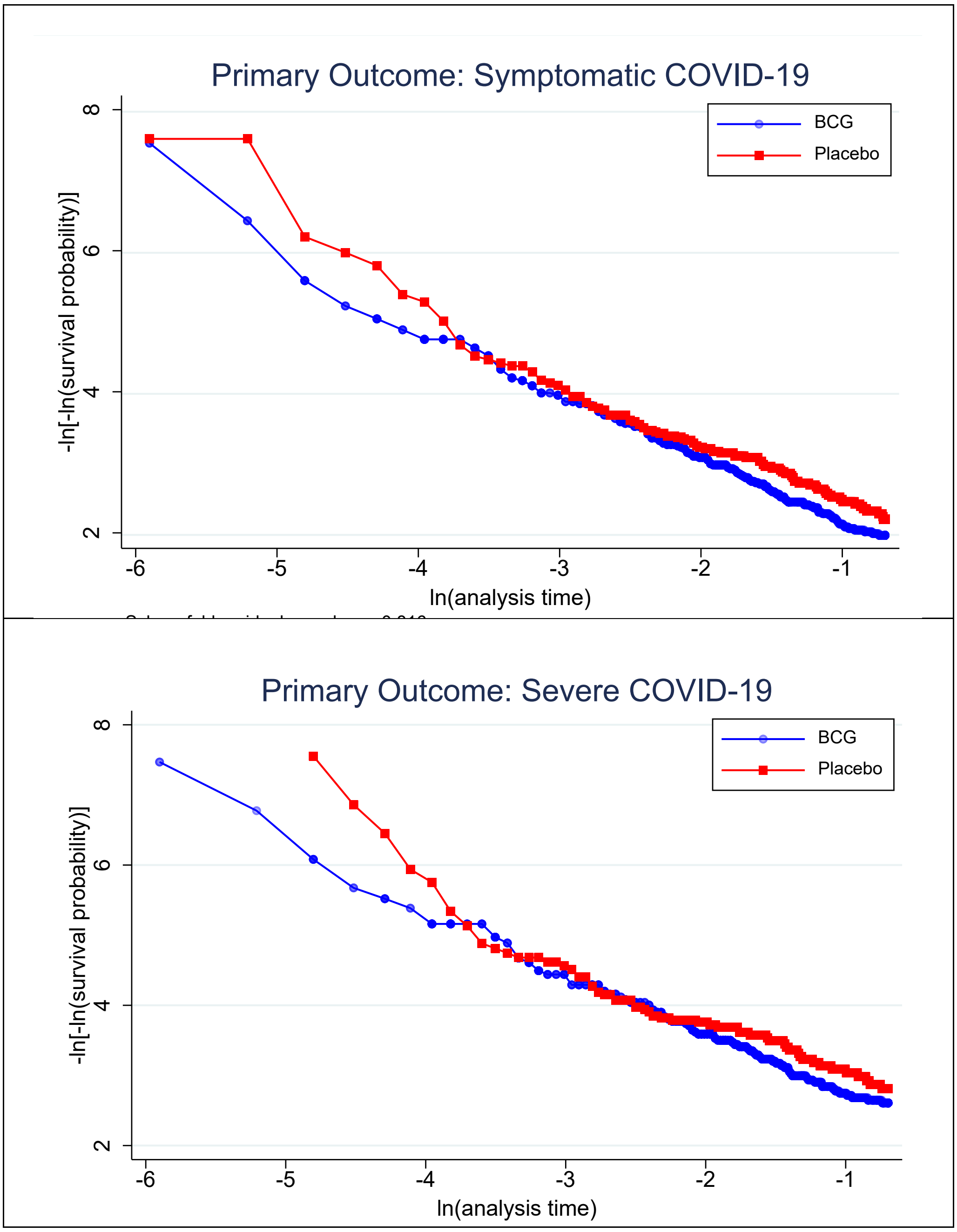

Table S18 Serious Adverse Events Occurring in the First Three Months of the BRACE Trial (Stage 2; Safety Population)

Sex	Vaccine group	Onset following vaccination	Description of adverse event	Type [a]	Treatment	Relationship to vaccine [b]	Severity [c]	Outcome
F	BCG	Day 4	Hospitalisation for vascular catheterisation; participant fell in pool and damaged portacath. Portacath had been inserted three years prior during management of breast cancer.	2	Hospitalisation: vascular catheterisation for removal of damaged portacath	(S)(M) Unrelated	Severe	Discharged home Day 5
F	BCG	Day 5	Discovered lump in right breast; referred to breast specialist; biopsy diagnosis of breast cancer.	7	Chemotherapy	(S)(M) Unrelated	Severe	Withdrawal from study on Day 12
F	BCG	Day 20	Hospitalisation for abdominal pain; gallstone blocking bile duct.	3	Hospitalisation: surgery to remove gallstones	(S)(M) Unrelated	Severe	Discharged home Day 25
F	BCG	Day 21	Hospitalisation for injection site abscess with pus discharge (approx. 80ml) and systemic symptoms. Plastics team review on readmission and immunology assessment.	3	Hospitalisation: IV flucloxacillin and gentamicin, IV fluids	(S)(M) Probable	Severe	Discharged home Day 37
M	BCG	Day 24	Hospitalisation for acute vomiting, diarrhoea and dehydration.	3	Hospitalisation: hydration	(S)(M) Unrelated	Severe	Discharged home Day 25
F	BCG	Day 29	Hospitalisation for cellulitis secondary to a cat scratch on right hand. No associated lymphadenopathy or fever. Injection site reaction healing well.	3	Hospitalisation: IV antibiotics	(S)(M) Unrelated	Severe	Discharged home Day 31
F	BCG	Day 37	Hospitalisation for epigastric pain.	3	Hospitalisation: symptomatic treatment	(S)(M) Unrelated	Severe	Discharged home Day 40
M	BCG	Day 43	Hospitalisation for COVID-19; mechanical ventilation in intensive care unit.	2	Hospitalisation: mechanical ventilation	(S)(M) Unrelated	Life-threatening	Discharged home Day 139
M	BCG	Day 46	Hospitalisation for COVID-19; mechanical ventilation in intensive care unit.	2	Hospitalisation: mechanical ventilation	(S)(M) Unrelated	Life-threatening	Discharged home Day 64
M	BCG	Day 54	First hospitalisation: overnight admission to intensive care unit following planned surgery for transurethral resection of the prostate and removal of wires from previously fractured patella.	4	Hospitalisation: intensive care unit, cardiac echocardiogram, right leg Doppler ultrasound.	(S)(M) Unrelated	Life-threatening	Discharged home Day 79
F	BCG	Day 63	Hospitalisation for psychiatric illness – depression.	2	Hospitalisation	(S)(M) Unrelated	Life-threatening	Discharged home Day 77
F	BCG	Day 65	Hospitalised for cardiac symptoms, related to underlying chronic disease.	3	Hospitalisation	(S)(M) Unrelated	Severe	Discharged home Day 68
M	BCG	Day 68	Hospitalisation for acute appendicitis.	3	Hospitalisation	(S)(M) Unrelated	Severe	Discharged home Day 69
F	BCG	Day 73	Hospitalisation for COVID-19; presented with shortness of breath and decompensated diabetes (ketoacidosis).	2	Hospitalisation: intensive care unit	(S)(M) Unrelated	Life-threatening	Discharged home Day 82
F	BCG	Day 78	Overnight hospitalisation in emergency department for Crohn's disease	3	Hospitalisation: faecal calprotectin test. Crohn's disease diagnosed on subsequent colonoscopy	(S)(M) Unlikely	Severe	Discharged home Day 79

M	BCG	Day 80	Second hospitalisation for fever and haematuria following transurethral resection of the prostate.	3	Hospitalisation: urinary tract ultrasound, septic work-up.	(S)(M) Unrelated	Severe	Discharged home Day 84
M	BCG	Day 86	Hospitalisation for appendicitis, with emergency surgery.	3	Hospitalisation: emergency surgery	(S)(M) Unrelated	Severe	Discharged home Day 88
F	BCG	Day 88	Hospitalisation for renal infection.	2	Hospitalisation	(S)(M) Unrelated	Severe	Discharged home Day 91
F	BCG	Day 89	Prolonged hospitalisation due to complication following endoscopic submucosal dissection for removal of a rectal carcinoma.	3	Hospitalisation: surgery for removal of carcinoma. No chemotherapy or radiotherapy requirement.	(S)(M) Unrelated	Severe	Discharged home on Day 91
F	BCG	Day 91	Hospitalisation for COVID-19 pneumonia.	3	Hospitalisation	(S)(M) Unrelated	Severe	Discharged home Day 96
F	BCG	Day 91	Hospitalisation for cough and shortness of breath; COVID-19 negative.	2	Hospitalisation	(S)(M) Unrelated	Severe	Discharged home Day 94

F	Placebo	Day 15	Hospitalisation for blunt cut injury to left hand (with left index finger paraesthesia) secondary to domestic knife incident.	3	Hospitalisation: exploratory surgery for neurological damage and carpal tunnel release.	(S)(M) Unrelated	Severe	Discharged home Day 16
M	Placebo	Day 21	Hospitalisation for COVID-19.	2	Hospitalisation: non-invasive ventilation	(S)(M) Unrelated	Life-threatening	Discharged home Day 28
F	Placebo	Day 25	Hospitalisation for COVID-19; mechanical ventilation in intensive care unit.	1	Hospitalisation: mechanical ventilation	(S)(M) Unrelated	Death	Death on Day 49
F	Placebo	Day 28	Hospitalisation for COVID-19.	2	Hospitalisation: non-invasive ventilation	(S)(M) Unrelated	Life-threatening	Discharged home Day 31
M	Placebo	Day 39	Hospitalisation for myocardial infarction.	3	Hospitalisation	(S)(M) Unrelated	Severe	Discharged home Day 42
F	Placebo	Day 72	Hospitalisation for femur fracture secondary to skate fall.	3	Hospitalisation: hip surgery (left femur)	(S)(M) Unrelated	Severe	Discharged home Day 78
M	Placebo	Day 82	Hospitalisation for dengue with complications.	2	Hospitalisation	(S)(M) Unrelated	Life-threatening	Discharged home Day 85
M	Placebo	Day 86	Hospitalisation for COVID-19; shortness of breath presentation.	2	Hospitalisation	(S)(M) Unrelated	Life-threatening	Discharged home Day 101
F	Placebo	Day 90	Hospitalisation for ankle fracture injury secondary to being run over.	3	Hospitalisation: ankle surgery (right tibia)	(S)(M) Unrelated	Severe	Discharged home Day 96

(S) = Site Investigator assessment
(M) = MCRI Sponsor assessment

[a] **SAE type = criteria for seriousness**
1. Resulted in death
2. Immediately life-threatening
3. Requires inpatient hospitalisation (i.e. minimum overnight admission that is non-elective).
4. Results in prolongation of existing hospitalisation
5. Results in persistent or significant disability/incapacity
6. Is a congenital anomaly/birth defect
7. In the medical judgment of the treating physician and/or investigator, it may jeopardise the participant or require intervention to prevent one of the above outcomes

[b] **Severity of SAE:**
- Severe (severe medically significant but not immediately life threatening)
- Life-threatening (immediately life-threatening)
- Death related to adverse event

[c] **Definition of relationship to the intervention**
- Unrelated (The AE is clearly NOT related to intervention)
- Unlikely (The AE is doubtfully related to the intervention)
- Possible (The AE may be related to the intervention)
- Probable (The AE is likely related to the intervention)
- Definite (The AE is clearly related to the intervention)

Table S19 Adverse Events of Interest Occurring in the First Three Months of the BRACE Trial (Stage 2; Safety Population)

Sex	Vaccine group	Onset following vaccination	Description of adverse event	Treatment	Relationship to vaccine *	Outcome
M	BCG	Day 2	Abscess	Oral flucloxacillin and drainage	Definite	Resolved Day 22
F	BCG	Day 5	Abscess	None required	Definite	Resolved Day 49
F	BCG	Day 5	Abscess	None required	Definite	Resolved Day 19
M	BCG	Day 7	Abscess	None required	Probable	Resolved Day 108
F	BCG	Day 14	Abscess	Analgesia and cooling	Definite	Resolved Day 57
F	BCG	Day 14	Abscess	Analgesia	Definite	Resolved Day 90
F	BCG	Day 17	Abscess	None required	Definite	Resolved Day 29
F	BCG	Day 17	Abscess	None required	Definite	Resolved Day 22
F	BCG	Day 20	Abscess	Surgical excision and amoxicillin/clavulanic acid	Definite	Resolved Day 82
F	BCG	Day 22	Abscess	None required	Definite	Resolved Day 43
M	BCG	Day 26	Abscess	None required	Definite	Resolved Day 30
F	BCG	Day 27	Abscess	ED presentation: flucloxacillin treatment	Definite	Resolved Day 115
F	BCG	Day 29	Abscess	None required	Definite	Resolved Day 34
M	BCG	Day 38	Abscess	Analgesia	Definite	Resolved Day 77

Representativeness of Study Participants

Table S20 Representativeness of Study Participants

**Category**	
Disease, problem, or condition under investigation	COVID-19
Special considerations related to	
Sex and gender	COVID-19 morbidity and mortality is higher in men
Age	COVID-19 morbidity and mortality increase steeply with age
Race or ethnic group	COVID-19 affects all populations. Some evidence suggests worse outcomes in minority racial and ethnic groups.
Geography	Reported prevalence and COVID-19-associated morbidity and mortality vary between countries but it is uncertain to what extent this reflects recognition, identification and reporting of cases, or national pandemic mitigation and management strategies.
Other considerations	None
Overall representativeness of this trial	There was a higher proportion of female than male participants, typical of trials recruiting in healthcare settings where the workforce is predominantly female. Less than 10% of participants were aged 60 years or over. Participants were recruited in 35 sites in 5 countries in Australia, Europe and South America.
